# A Microfluidic Multisize Spheroid Array for Multiparametric Screening of Anticancer Drugs and Blood–Brain Barrier Transport Properties

**DOI:** 10.1002/advs.202004856

**Published:** 2021-03-24

**Authors:** Christoph Eilenberger, Mario Rothbauer, Florian Selinger, Anna Gerhartl, Christian Jordan, Michael Harasek, Barbara Schädl, Johannes Grillari, Julian Weghuber, Winfried Neuhaus, Seta Küpcü, Peter Ertl

**Affiliations:** ^1^ Faculty of Technical Chemistry Vienna University of Technology Getreidemarkt 9 Vienna 1060 Austria; ^2^ Karl Chiari Lab for Orthopaedic Biology Department of Orthopedics and Trauma Surgery Medical University of Vienna Währinger Gürtel 18‐20 Vienna 1090 Austria; ^3^ AIT Austrian Institute of Technology GmbH Center Health and Bioresources Competence Unit Molecular Diagnostics Giefinggasse 4 Vienna 1210 Austria; ^4^ Ludwig‐Boltzmann‐Institute for Experimental and Clinical Traumatology Donaueschingenstraße 13 Vienna 1200 Austria; ^5^ Institute for Molecular Biotechnology Department of Biotechnology University of Natural Resources and Life Sciences Muthgasse 18 Vienna 1190 Austria; ^6^ School of Engineering University of Applied Sciences Upper Austria Stelzhamerstraße 23 Wels 4600 Austria; ^7^ FFoQSI GmbH‐Austrian Competence Centre for Feed and Food Quality Safety and Innovation Technopark 1C Tulln 3430 Austria; ^8^ Institute of Synthetic Bioarchitectures Department of Nanobiotechnology University of Natural Resources and Life Sciences Vienna, Muthgasse 11 Vienna 1190 Austria

**Keywords:** anticancer drugs, blood‐brain barrier, in vitro tests, microfluidics, multicellular spheroids

## Abstract

Physiological‐relevant in vitro tissue models with their promise of better predictability have the potential to improve drug screening outcomes in preclinical studies. Despite the advances of spheroid models in pharmaceutical screening applications, variations in spheroid size and consequential altered cell responses often lead to nonreproducible and unpredictable results. Here, a microfluidic multisize spheroid array is established and characterized using liver, lung, colon, and skin cells as well as a triple‐culture model of the blood‐brain barrier (BBB) to assess the effects of spheroid size on (a) anticancer drug toxicity and (b) compound penetration across an advanced BBB model. The reproducible on‐chip generation of 360 spheroids of five dimensions on a well‐plate format using an integrated microlens technology is demonstrated. While spheroid size‐related IC_50_ values vary up to 160% using the anticancer drugs cisplatin (CIS) or doxorubicin (DOX), reduced CIS:DOX drug dose combinations eliminate all lung microtumors independent of their sizes. A further application includes optimizing cell seeding ratios and size‐dependent compound uptake studies in a perfused BBB model. Generally, smaller BBB‐spheroids reveal an 80% higher compound penetration than larger spheroids while verifying the BBB opening effect of mannitol and a spheroid size‐related modulation on paracellular transport properties.

## Graphical abstract

A microfluidic platform is presented that builds on the advancement of a spheroid‐on‐a‐chip technology that accommodates 360 spheroids of five different dimensions on a microtiter plate format supporting a range of human tissue models. Thus, this platform closes an essential technological gap, enabling rapid and easy production of spheroids of defined sizes for pharmaceutical screening in a standardized and reproducible manner.

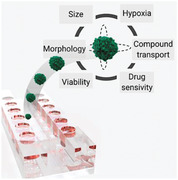

## 1 Introduction

The costs of drug development increase exponentially, starting at the late stage of preclinical testing using in vivo models followed by lengthy clinical trials.^[^ ^1^ ^]^ In addition to the increased financial burden, the majority of initially identified compounds with potential health benefits are steadily eliminated during clinical phase periods one, two, and three. This high drug failure rate in the pharmaceutical development cycle has mainly been attributed to the lack of predictability in the early preclinical phase testing using standard in vitro and in vivo models. Similar situations have also been reported by other industries that regularly develop new chemicals for consumer use, including cosmetics, agro‐food, and consumer goods.^[^ ^2^ ^]^ To improve the predictability of preclinical in vitro models, recent efforts of pharmaceutical companies are based on implementing complex 3D biological systems such as multicellular spheroid and organoid technologies. Since multicellular spheroid systems are able to mimic human (patho)physiologies, they are considered a promising alternative to bridge the gap between preclinical tests and in vivo outcomes by eliminating unsuitable agents early on.^[^ ^3^, ^4^ ^]^ As a result, the application of multicellular spheroid systems in industrial settings can potentially lead to significantly lower pharmaceutical development costs by shortening development time, providing meaningful and representative test results.^[^ ^5^ ^]^

Despite the many potentials of using multicellular spheroid systems as advanced in vitro models, including 1) more predictive and reproducible toxicity and efficacy tests, 2) early exclusion of drug candidates in the drug development pipeline, 3) the possibility to perform substance testing on relevant human disease models, and 4) a reduction of animal studies, thus following the 3R principle (e.g., replacing, reducing, refining animal testing),^[^ ^6^ ^]^ some distinct limitations still remain. The main drawback of using complex multicellular spheroid systems in the drug development process is the lack of standardization and harmonization across the industry leading to significant variations in spheroid morphologies,^[^ ^7^, ^8^ ^]^ cell numbers and ratios used, medium compositions, and cultivation/assay times,^[^ ^9^ ^]^ which essentially eliminates a meaningful comparison between different end‐users and laboratories. Recently, we have shown that spheroid age variations and lack of reproducible uniformity impact the outcome of drug delivery and efficacy studies,^[^ ^10^ ^]^ thus preventing the integration of this promising technology into mainstream drug discovery pipelines. It is important to highlight that the generated size of multicellular spheroids, which ranges in the hundreds of microns in diameter (e.g., 100 to 1000 µm), can be considered a primary critical parameter that influences gradient distributions of oxygen, growth factors, nutrition (e.g., sugar, peptides, proteins), ions, and pH as well as guiding the elimination of metabolic wastes inside the spheroid, thus tissue size heavily impacts all aspects of cellular functions.^[^ ^11^, ^12^, ^13^ ^]^ Taken into a pharmaceutical context, the altered mass transport properties in differently sized spheroids further modulate penetration, distribution, and retention of drugs directly and impact spheroid (size‐related) drug response.^[^ ^12^ ^]^ As an example, larger tumor spheroids are known to display higher chemoresistance due to i) increased contact‐mediated resistance, ii) exclusion of drugs, and iii) their content of proliferating and hypoxic cells resulting from more pronounced nutrient and oxygen gradients.^[^ ^14^, ^15^ ^]^ Additional reports indicated spheroid size‐related biological effects such as altered protein production as albumin secretion,^[^ ^16^ ^]^ amount of cancer stem cell accumulation in tumor spheroids,^[^ ^17^ ^]^ shifts in differentiation pattern in human embryoid bodies,^[^ ^18^ ^]^ as well as cell‐type‐specific tissue stiffness variations (e.g., loose vs tight cell aggregates).^[^ ^19^ ^]^

To date, a number of methods for multicellular 3D spheroid generation exist, including nonadhesive surfaces,^[^ ^20^, ^21^ ^]^ spinner flasks,^[^ ^22^ ^]^ scaffold supports,^[^ ^23^ ^]^ acoustic tweezers,^[^ ^24^ ^]^ hanging drops,^[^ ^25^, ^26^ ^]^ microwells,^[^ ^27^, ^28^, ^29^ ^]^ as well as various microfluidic devices.^[^ ^30^, ^31^ ^]^ Among these, only the hanging drop technology and microwell‐based methods combined with precision fabrication techniques such as lithography, 3D‐printing, and computerized numerical control milling can achieve homogenous spheroids with controllable sizes.^[^ ^26^ ^]^ Despite their ability to generate uniform spheroid sizes, these techniques are highly laborious and, at times, technical challenging, thus limiting their scalability. Alternatively, in recent years, microfluidics technology has been used to produce chip designs capable of controlling spheroid size and growth dynamics.^[^ ^27^, ^32^, ^33^, ^34^, ^35^ ^]^ Unfortunately, most microfluidic spheroid technologies still lack automatic generation and cultivation of 3D spheroids as well as the formation of different‐sized spheroids on a single chip‐platform, which is needed to account for size‐dependent compound toxicities, drug responses, and biological phenomena. Consequently, to meet the growing demand for medium‐throughput and high‐content multicellular spheroid systems, next‐generation microfluidic devices need to offer 1) optimal tissue culture conditions including tight control of medium composition and gas exchange, 2) simple and robust cell loading procedures, 3) parallel spheroid production of different sizes, and 4) dynamic medium perfusion as well as 5) simple operation with reproducible tissue maintenance.^[^ ^36^ ^]^ To address these challenges, we have developed a microfluidic multisize spheroid array capable of culturing 3D multicellular spheroids with high reproducibility in medium‐to high‐throughput formats using a wide range of different tissue types.

**Figure 1:**
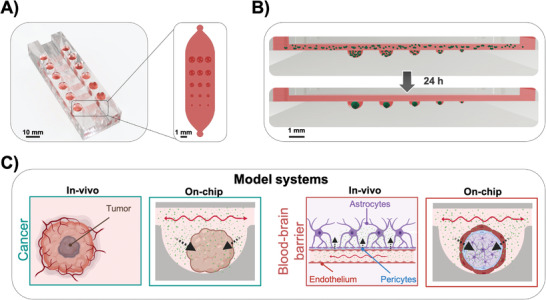
A) A cutaway rendering of the microfluidic spheroid array showing six microfluidic channels, each containing 15 spheroids with five different sizes and respective medium reservoirs, which can be addressed by multichannel pipettes. B) Workflow of parallel on‐chip spheroid generation within 24 h. C) Overview of the established cell model systems, including spheroid tumor models and 3D BBB models for pharmaceutical screening applications. Arrows indicate diffusion of anticancer drugs or active and passive transport across the BBB in vivo and on the chip.

In this study, we demonstrate the reliable and reproducible generation of 90 multiple‐sized spheroids on a single chip and the formation of 360 spheroids on a “microtiter plate”‐based platform layout as shown in Figure S1 (Supporting Information). To ensure medium‐throughput capability, gravity‐driven perfusion is selected, whereby flow velocities are adjusted by an embedded flow restrictor in combination with tilting angle and speed of a conventional laboratory rocker. Additionally, medium reservoirs are arranged at a 9 mm pitch to be compatible with standard multichannel pipettes for 96‐well microtiter plates. The microfluidic multisize spheroid array shown in **Figure** **1**A is therefore comprised of three main components: i) six microfluidic culture channels in a standard 96‐well plate footprint each containing 15 individual microwells with five diameters of 1000, 900, 700, 500, and 300 µm, ii) perfusion connectors incorporated into the cover layer that interconnect the inlets and outlets of the channels with medium reservoirs and air bubble traps, and iii) a pair of medium reservoirs for each culture channel that can be filled using a multichannel pipettor to enable straightforward and simple cell seeding as well as facile retrieval of supernatant and cellular material. A rendered cutaway of the platform is seen in Figure S2 (Supporting Information) and shows the different microfluidic layers, which are constructed using soft lithography from polydimethyl siloxane (PDMS). The main feature of the microfluidic multi‐sized spheroid array highlighted in Figure 1B, however, is the integration of different‐sized microwells of defined semispherical geometry capable of reliably trapping increasing cell numbers. Spontaneous cell aggregation within 24 hours is accomplished by surface modification using a biocompatible low‐adhesive 2‐(methacryloyoxy)ethyl phosphorylcholine (MPC) polymer. Initial performance evaluation of our microfluidic multisize spheroid array biochip includes a comparison of morphometric and metabolic parameters using four different well‐established cancer and noncancerous cell lines cultured under continuous perfusion for 12 d. Practical applications of the microfluidic multicellular spheroid technology involve a) an anticancer screening approach and b) a blood–brain barrier drug penetration study as outlined in Figure 1C. Here, we demonstrate that our multisize spheroid platform is compatible with the standard software and hardware of a high‐content live‐cell imaging system by analyzing spheroid size, morphology, cellular activity, hypoxia levels, transport of fluorescent‐labeled compounds, and drug‐dose responses.

In summary, our study focuses on the establishment of a variety of in vitro spheroid‐based spheroid models used i) to optimize cell culture conditions, including seeding densities and coculture cell ratios, ii) to evaluate two clinically relevant anticancer drugs for therapy optimization studies and iii) to investigate active and passive transport across the blood–brain barrier. Thus, our microfluidic multisize spheroid array closes a critical technological gap, enabling rapid and easy production of spheroids of defined size and cell types.

## 2 Results and Discussion

### 2.1 Identification of Best Microwell Dimensions for the Formation of Multisized Spheroids

**Figure 2:**
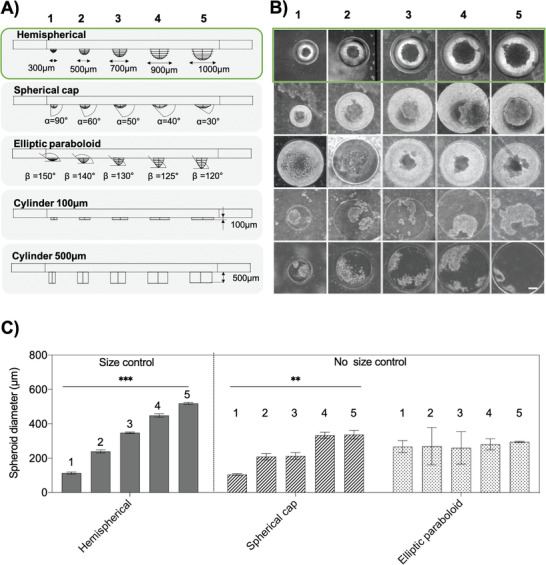
A) Cross‐sections of CNC milled microwells of different geometries, including hemispherical microlenses, spherical caps, elliptic paraboloids, and cylinders. B) Bright‐field micrographs of HepG2 spheroids after three days of on‐chip cultivation. Scale bar, 100 µm. C) Optimization of microwells by evaluating the controllability of HepG2 sizes in terms of different microwell geometries, *n* = 3–6 ± SD. Statistical analysis was performed using the mixed‐effects analysis (**p < 0.0021, ***p < 0002).

Although cell trapping in microcavities is by far the most popular technique to generate spheroids in microfluidic devices, this approach results in high variability of spheroid quality, thus hindering standardization and comparability. To evaluate whether a specific geometric feature allows precise control over the formation of reproducible, uniformly sized and single multicellular spheroids of defined dimensions, various well shapes and geometries were investigated. In total, five geometries with varying dimensions as shown in **Figure** **2**A, including flat‐bottom wells (cylinder of 100 and 500 µm depth), spherical caps, elliptic paraboloids, and hemispheres were evaluated on their ability to generate uniform spheroids reproducibly. After 3 d postseeding, the total number of individual spheroids formed, spheroid roundness, center‐to‐center distance, and size controllability of HepG2 spheroids were compared using bright‐field micrographs as depicted in Figure 2B (see also Figure S3, Supporting Information). Results of this comparative study are shown in **Table** **1**, indicating that only hemispherical dimensions using a microlens design fostered the formation of single spheroids in every microwell diameter (total of 15 wells). In contrast, wells with sharper or flatter curvatures and cylindrical shapes revealed a higher probability of multiple spheroid formations in each cavity, thus decreasing accuracy. These results clearly eliminate flat‐bottom shapes and favor round‐bottom shapes to ensure reliable formation of a single spheroid within each well. To assess the influence of round‐bottom microwell shapes on the quality of spheroid morphology in more detail, each spheroids’ roundness was determined using cylinders (100 and 500 µm depth), a spherical cap, and a hemisphere shape. Results reveal that both the hemispherical‐ and the spherical cap‐shapes generate highly reproducible, round HepG2 spheroids in each well diameter, exhibiting an overall roundness factor of 0.95 ± 0.04 and 0.94 ± 0.03, respectively. In contrast, spheroids located in flat‐bottom cylinder shapes with depths of 100 and 500 µm exhibited decreased roundness with factors of 0.57 ± 0.07, 0.49 ± 0.07 for each respective shape. Additionally, spheroids formed in elliptical paraboloid‐shaped wells revealed comparable roundness factors of hemispherical and cap‐shaped wells only at wider polar angles of 150° and 140°. Interestingly, with an increasing polar angle, a reduction in roundness below 0.9 was observed. It is important to note that only spheroids with a roundness above 0.9 are considered as regular spherical‐shaped spheroids as described in literature.^[^ ^37^ ^]^ This means that both of the cylindrical shapes generated irregular, noncircular HepG2 spheroids.

**Table 1:** Optimization of microwells by evaluating a number of spheroids per well, spheroid roundness, and spheroid center‐to‐microwell center‐distances. Data are expressed as mean value ± SD for *n* = 3. Underlined values are considered as the most optimal shape parameter

Parameter		Hemispherical	Spherical cap	Elliptic paraboloid	Cylinder 100 µm	Cylinder 500 µm
Spheroid number per well (optimum: 1.0)	1	1.0 ± 0.0	1.0 ± 0.0	1.0 ± 0.0	2.3 ± 0.6	1.3 ± 0.6
	2	1.0 ± 0.0	1.3 ± 0.3	1.0 ± 0.0	2.7 ± 1.2	1.7 ± 0.6
	3	1.0 ± 0.0	1.0 ± 0.0	1.0 ± 0.0	6.3 ± 2.1	2.7 ± 0.6
	4	1.0 ± 0.0	1.3 ± 0.5	1.3 ± 0.6	6.0 ± 1.7	3.7 ± 1.2
	5	1.0 ± 0.0	1.7 ± 0.6	1.0 ± 0.0	7.7 ± 1.5	3.0 ± 1.0
Roundness per well [AU] (optimum ≥ 0.9)	1	1.0 ± 0.0	0.9 ± 0.0	0.9 ± 0.0	0.5 ± 0.0	0.5 ± 0.2
	2	1.0 ± 0.1	0.9 ± 0.0	1.0 ± 0.0	0.6 ± 0.1	0.5 ± 0.0
	3	0.9 ± 0.0	0.9 ± 0.0	0.8 ± 0.0	0.6 ± 0.1	0.5 ± 0.1
	4	0.9 ± 0.0	0.9 ± 0.0	0.8 ± 0.0	0.7 ± 0.1	0.5 ± 0.0
	5	0.9 ± 0.0	1.0 ± 0.0	0.7 ± 0.0	0.5 ± 0.0	0.5 ± 0.1
Center‐to‐center distance per well [µm] (optimum = 0 µm)	1	5.4 ± 6.6	47.3 ± 25.7	27.3 ± 19.1	76.1 ± 26.4	89.3 ± 25.60
	2	16.3 ± 8.0	55.4 ± 33.9	23.3 ± 17.7	138.4 ± 54.1	121.6 ± 31.7
	3	18.5 ± 7.5	31.7 ± 0.1	4.8 ± 1.7	228.2 ± 54.8	244.7 ± 26.3
	4	10.3 ± 2.0	40.0 ± 5.5	26.3 ± 29.6	280.4 ± 146.5	316.5 ± 59.6
	5	10.1 ± 6.3	53.9 ± 32.3	23.9±20.9	376.0 ± 113.6	410.0 ± 12.7

Final microfluidic microwell array evaluation involved reliable localization of spheroids and size control measures, which are essential aspects for automation, signal processing, and image analysis in medium‐ to high‐throughput screening applications. While flat bottom layouts yielded the highest center‐to‐center distances (e.g., from 410 µm to 89 µm in 500 µm cylinders), hemispherical wells showed the lowest center‐to‐center variations with distances of 10.1 ± 6.3, 10.3 ± 2.0, 18.5 ± 7.5, 16.3 ± 8.0, and 5.4 ± 6.6 µm from the largest (1000 µm) to the smallest (300 µm) cavity. Based on the results above, only hemispherical, spherical cap, and elliptic paraboloid shapes were evaluated for spheroid size controllability in subsequent experiments. Results in Figure 2C demonstrate that only hemispherical cavities/microwell shapes are able to reliably generate spheroids of increasing sizes in a linear fashion exhibiting diameters of 113.1 ± 6.3, 239.3 ± 9.4, 347.5 ± 4.7, 448.4 ± 10.2, and 519.2 ± 6.4 µm. In turn, elliptical paraboloid‐ and spherical cap‐shaped cavities resulted in an irregular and less controllable spheroids formation (no linear increase and correlation). In summary, our highly optical, transparent hemispherical microwell design based on “microlens” dimensions is ideally suited to generate spheroids of defined sizes, geometric features, and similar locations within a microfluidic spheroid array.

### 2.2 Characterization of Dynamic Culture Conditions Using a Bidirectional Hydrostatic Flow

**Figure 3:**
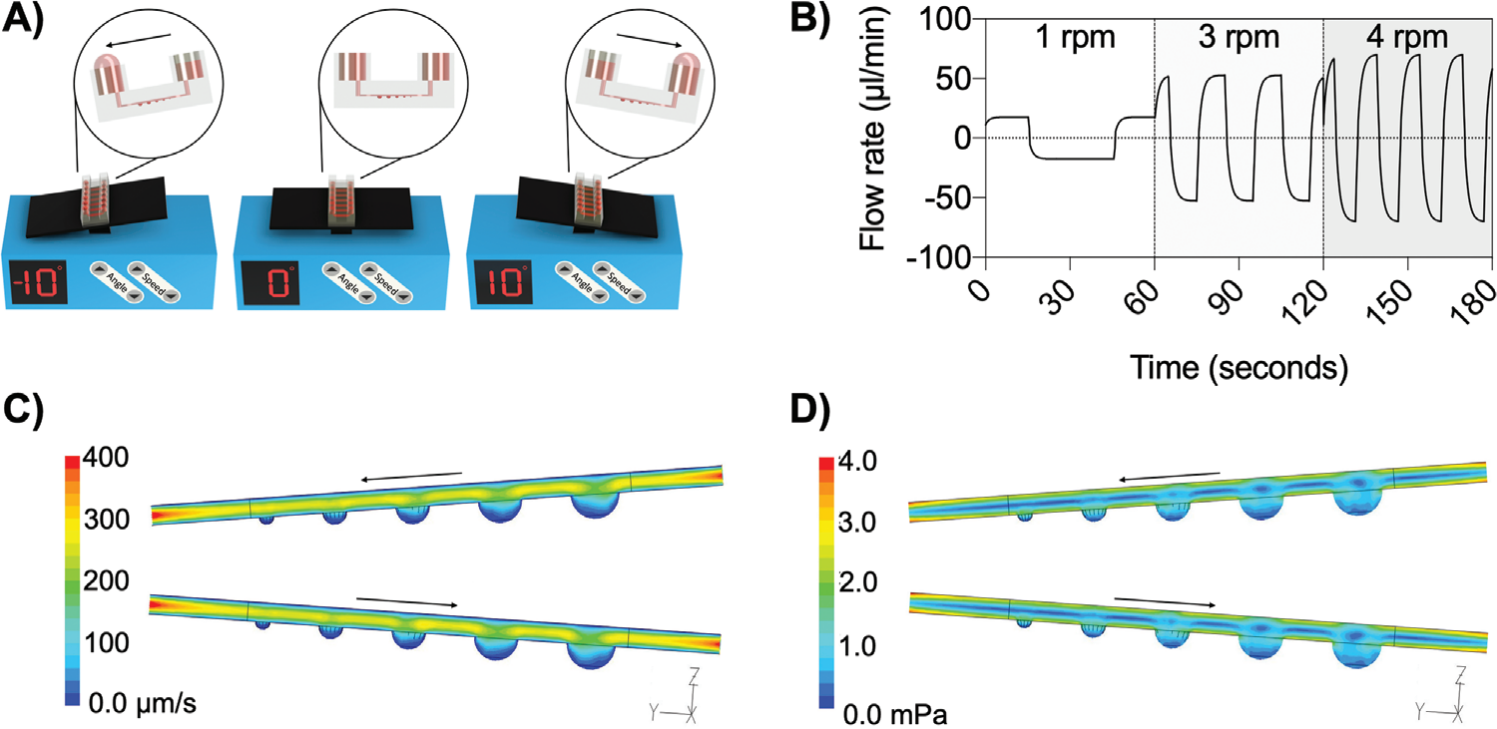
A) Tilting schemes of the microfluidic spheroid array system by gravity‐driven flow. B) Flow profiles at tilting speeds of 1, 3, and 4 rpm at a fixed angle of 1°. C) Flow velocity and D) shear stress at a constant tilting speed of 1 rpm at a tilting inclination angle of 1° in spheroid culture channels.

Supply and continuous perfusion of cell culture medium were achieved by gravity‐induced bidirectional fluid circulation using an automated tilting motion of the microfluidic multisized spheroid array, as shown in **Figure** **3**A. Some advantages of this pumpless‐flow strategy are the ability i) to adjust flow profiles by modifying the tilting angle and speed, ii) to reduce bubble formation, and iii) to reproduce pulsating nature of blood circulation, as depicted in Figure 3B. Since gravity‐driven perfusion results in rapid flow profile changes within the microchannel network, flow restrictors are additionally embedded underneath each medium reservoir to increase the hydraulic resistances of the microfluidic channel, thus passively controlling flow velocities. To estimate fluid velocities and shear forces of the continuous bi‐directional microfluidic flow under different operating conditions, computational fluid dynamics (CFD) simulations and experiments were performed. Initially, flow rates (at a period of 60 s) were determined in silico to assess three different gravity‐flow protocols using a fixed tilting angle of 1° in the presence of increasing tilting speeds. Results of our fluid dynamics study are shown in Figure 3B, where reproducible net flow rates of 0.4, 1.0, and 2.1 µL min^−1^ were estimated using tilting speeds of 1, 3, and 4 rpm, respectively. An additional increase of pulsation rates from 0.01 to 0.05 Hz yielded maximal flow rates ranging from 17.4 to 70 µL min^−1^. To validate these computational results, fluid column heights in the reservoirs were measured at defined tilting angles to calculate hydrostatic pressures and resulting flow velocities. As an example, **Table** **2** shows no significant differences of simulated versus measured flow rates at increasing tilting angles and a fixed tilting speed of 1 rpm, which points to the ability to reliably control flow velocities between 15.7 ± 9.2 and 176.0 ± 33.9 µL min^−1^. It is important to note that this elevated flow regime provided homogenous distribution of cell suspension during cell loading and trapping in microwells and efficiently removed nontrapped cells in the antiadhesive coated microchannel network. To further estimate generated flow rates and shear forces present inside the cavities where spheroids reside, additional CFD simulations were performed. Results of 3D CFD simulations (see Figure 3C,D) reveal a 75% to 80% reduction in fluid velocity of 37.9 ± 16.1 µm s^−1^ in the microwells and a shear stress reduction to 1.4 ± 0.2 mPa (at a tilting angle of 1° and 1 rpm). Moreover, fluid streamlines fully enveloped the entire spheroid without indication of turbulences, thus pointing at an efficient medium turnover inside the growth compartment, as shown in Figure S4 (Supporting Information). Notably, microwell flow velocity and shear stress increased to 72.06 ± 30.8 µm s^−1^ and 2.9 ± 0.4 mPa as well as 109.2 ± 46.6 µm s^−1^ and 4.2 ± 0.6 mPa with rising tilting frequencies of 3 and 4 rpm, when keeping tilting angle constant (data not shown). These results demonstrate that pumpless gravity‐driven flow is able to tune flow velocities inside the cavities and spheroids, which is needed to identify optimum cell culture conditions.

**Table 2:** Experimental versus in silico data of maximum flow rates as a function of tilting angles at a constant frequency of 1 rpm. Data are expressed as mean value ± SD for *n* = 6

	Tilting angle				
Flow rate [µL min^−1^]	1°	3°	5°	7°	10°
Experimental	15.7 ± 9.2	57.6 ± 22.9	90.0 ± 28.6	126.3 ± 33.1	176.0 ± 33.9
Simulation	17.9 ± 0.0	53.8 ± 0.08	89.5 ± 0.1	125.1 ± 0.1	175.4 ± 0.2

### 2.3 On‐Chip Generation of Multitissue Spheroids and Characterization of Linear Size‐Control Strategy

**Figure 4:**
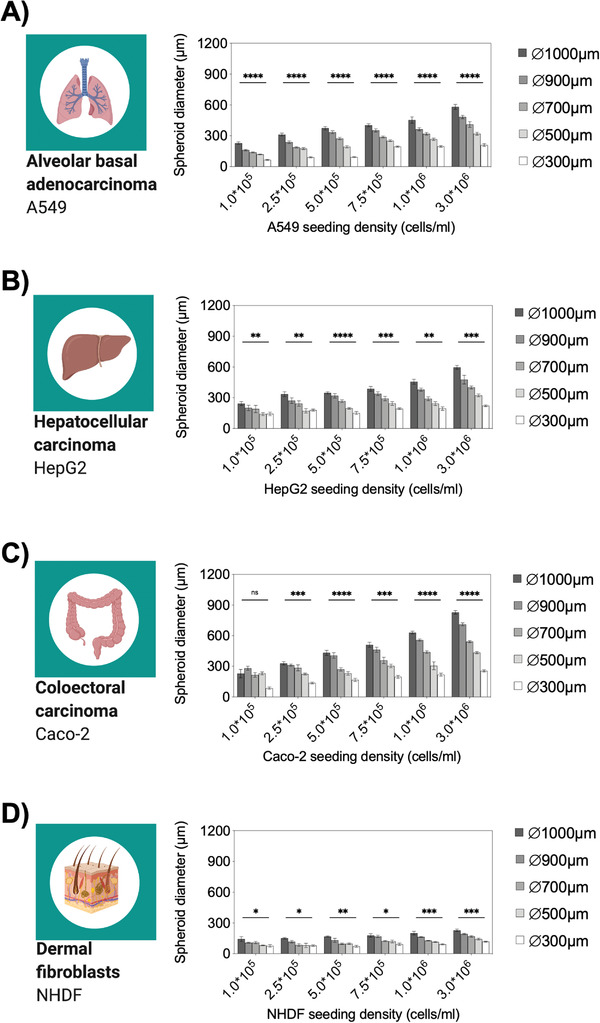
Analysis of spheroid diameters of A) A549, B) HepG2, C) Caco‐2 and D) NHDF spheroids at different initial seeding densities after 3 d postseeding under continuous perfusion in the microfluidic spheroid array device, *n* = 6–9 ± SD. Statistical analysis was performed using mixed‐effects analysis (*p < 0.0332, **p < 0.0021, ***p < 0.0002, ****p < 0.0001).

Since differences in tissue types and growth can result in inconsistent assessments between multiple spheroid cell‐line cultures, initial testing of seeding densities is crucial to describe the cell type‐specific behavior regarding spheroid size and cellular growth. One important aspect of those evaluations is the capability to assess direct relationships between initial cell seeding concentrations and spheroid sizes as well as linear spheroid size separation to ensure a broad range of dimensions on one chip. To evaluate spheroid growth rates in terms of diameter and spheroid size separation under continuous bidirectional perfusion, a panel of standardized cancer cell lines and human fibroblasts were recorded over a 12 d incubation period. **Figure** **4** shows measured spheroid diameters after 3 d in culture using lung, liver, colon, and skin cell cultures in the presence of increasing seeding densities to shed light on the relationship between initial cell seeding concentrations and spheroid sizes. Interestingly, cell type‐dependent spheroid diameters were already obtained after 3 d in on‐chip culture ranging from a minimum to a maximum diameter of 66. 2 ± 12.6 µm to 581.4 ± 58.4 µm for lung (A540), 142.6 ± 37.6 µm to 596 ± 50.5 µm for liver (HepG2), 86.2 ± 20.0 µm to 828.7 ± 49.5 µm for colon (Caco‐2) and 75.6 ± 30.3 µm to 229.2 ± 27.1 µm for skin (NHDF) spheroids. This means that by varying initial cell seeding densities a) an extensive range of spheroid sizes can be reliably generated, and b) cell line‐specific growth differences can be readily evaluated using our microfluidic multisize spheroid array. For instance, looking at lung A549 spheroid growth rates revealed that smaller spheroids exhibited a substantially larger size change over a 12 d cultivation period of 45% in 500 µm diameter wells than the 30% size expansion obtained with spheroids grown in 1000 µm diameter wells (see also Figure S5, Supporting Information). In turn, liver (HepG2) and colon (Caco‐2) spheroids exhibited a well diameter‐independent increase of size of approximately 50% and 30%, respectively. In contrast to lung and colon epithelial cells, dermal fibroblast spheroids showed a tendency to get more compact over time where spheroid diameters in 1000, 900, 700, 500, and 300 µm wells decreased by 20.6 ± 9.9%, 16.3 ± 9.5%, 17.6 ± 6.6%, 31.0 ± 12.1%, and 24.6 ± 15.8%, respectively.

As a first practical application of the microfluidic multisize spheroid array, a seeding density optimization study was conducted using a one‐way ANOVA and linear regression analyses to evaluate growth differences between the different tissue spheroid models. Initial ANOVA results showed significant differences among all evaluated seeding densities with calculated *p*‐values between *p* < 0.0332 and *p* < 0.0001, which pointed at a reliable spheroid generation of 270 spheroids in all chips. Next, optimal seeding protocols for increasing well sizes were evaluated on day three using linear regression analysis. **Table** **3** lists the calculated *R* ^2^‐values of each replicate value that indicate a linear trend with increasing seeding densities. This means that optimal seeding densities in terms of statistical significance and spheroid‐to‐well linearity were obtained at concentrations of 3 × 10^6^ cells mL^−1^ for HepG2, Caco‐2, and NHDF spheroids as well as 1 × 10^6^ cells mL^−1^ for A549 spheroids. As a consequence of these results, the above‐optimized seeding protocols were used for all subsequent experiments. Interestingly, individual slopes of the spheroid size separation can be tailored by simply adjusting initial seeding densities, thus enabling on‐demand spheroid size generation depending on initial seeding densities and microwell sizes.

**Table 3:** Linear regression analysis and goodness‐of‐fit (*R* ^2^) values of generated sizes after 3 d postseeding of A549, HepG2, Caco‐2, and NHDF spheroids in respect to initial seeding densities Statistical significance of respective slopes was determined by analysis of covariance (ANCOVA). Data are expressed as mean value ± SD for *n* = 6. Underlined values are considered as the most optimal seeding density

	Seeding density [cells mL^−1^]						
Cell line	1.0 × 10^5^	2.5 × 10^5^	5.0 × 10^5^	7.5 × 10^5^	1.0 × 10^6^	3.0 × 10^6^	*p*‐value of slopes
A549	0.8336	0.8388	0.8251	0.8725	0.8938	0.8747	*P* < 0.0001
HepG2	0.2701	0.4697	0.8278	0.6739	0.7988	0.8345	*P* < 0.0001
Caco‐2	0.1977	0.7552	0.7915	0.8140	0.9175	0.9662	*P* < 0.0001
NHDF	0.3463	0.3968	0.5596	0.5502	0.7430	0.7935	*P* = 0.1387

### 2.4 Multiparametric Monitoring of Multitissue Spheroids On‐Chip

**Figure 5:**
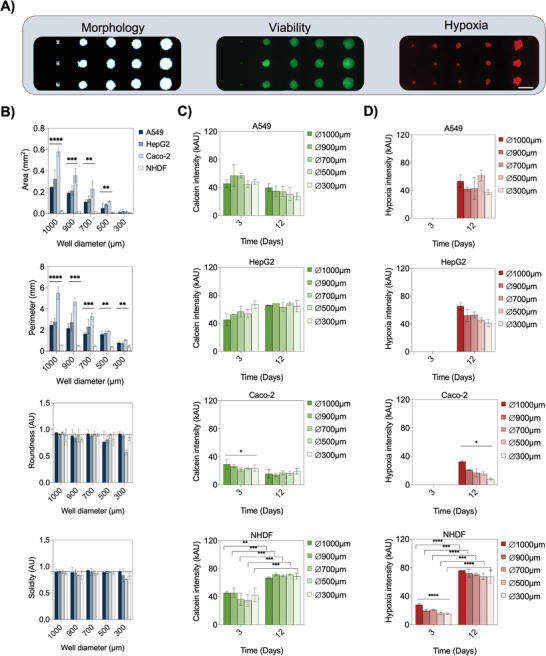
A) Representative bright‐field and fluorescent micrographs to evaluate morphology, intracellular esterase‐activity, and hypoxia during cultivation of spheroids in the microfluidic array. Scale bar, 1 mm. B) Morphometric analysis of area, perimeter, roundness, and solidity of A549, HepG2, Caco‐2, and NHDF spheroids with different sizes, *n* = 3 ± SD. Statistical analysis was performed using the one‐way ANOVA (*p < 0.0332, **p < 0.0021, ***p < 0002, ****p < 0.0001). C) Calcein and D) Hypoxia fluorescence intensities of the four cell lines at day 3 and 12 postseeding on‐chip of different sizes, as indicated in each graph, *n* = 3 ± SD. Statistical analysis was performed using one‐way ANOVA and Holm‐Sidak's multiple comparisons test (*p < 0.0332, **p < 0.0021, ***p < 0.0002, ****p < 0.0001).

Using the above‐optimized cell seeding densities for the five tissue types, time‐resolved images of individual spheroids were taken to investigate morphology changes, esterase activity shifts, and hypoxia occurrence in the next set of experiments (see **Figure** **5**A).To validate the spheroid quality of generated lung (A549), colon (Caco‐2), liver (HepG2), and skin (NHDF) spheroid cultures according to optimized seeding protocols, spheroid area, perimeter, roundness, and solidity were tested in detail to determine cell‐type‐specific morphological differences. Results in Figure 5B show significant changes of spheroid areas among presented cell lines and microlens diameters in the range from 0.005 to 0.6 mm^2^ and a direct proportional linear decrease with well diameter for A549, Caco‐2, and HepG2 spheroids. In turn, NHDF spheroids showed no significant area change in all microwell sizes. In the next step, individual spheroid perimeters were determined to quantify spheroid surface structure and smoothness. Here, significant variations in the topographic structures were found between all four cell lines, where Caco‐2 cells revealed the most unregular morphologies as indicated by perimeters in a range of 3.3 ± 0.3 mm. Interestingly, A549 lung and HepG2 liver cells displayed similar perimeters of 1.6 ± 0.2 and 2.3 ± 0.7 mm, respectively. Similar results were obtained in other well dimensions too. An alternative to size‐related spheroid quality parameter, roundness and solidity of spheroids determines the ability to form tight well‐defined round cell aggregates. Results shown in Figure 5B indicate the absence of significant roundness and solidity differences for all cell lines, thus pointing at the generation of stable and reproducible spheroids for various cell lines and tissue types.

To demonstrate that the platform's capability of performing functional fluorescent‐based assays in a size‐and cell type‐specific manner, we next monitored time‐resolved changes of esterase activity and hypoxia levels on‐chip. A panel of the previously characterized cell lines was cultivated and evaluated on‐chip with metabolic indicators using calcein‐AM as an intracellular esterase‐activity sensing solution and a reversible fluorogenic hypoxia reagent that responds to the low oxygen environment in the cell. Due to the high permeability of calcein‐AM, no size‐dependent differences in intracellular esterase activity were determined, except for Caco‐2 cells at day three (*P* = 0.0295), as shown in Figure 5C. However, differences in overall fluorescence intensity values, thus esterase activities, were cell‐line specific with significantly lower levels observable for Caco‐2 cells compared to the other cell lines. In detail, A549, HepG2, and NHDF spheroids showed mean intensities of 50.5 ± 8.8, 54.3 ± 8.9, and 41.0 ± 8.0 kAU after 3 d postseeding respectively, in contrast to significantly lower (*P* < 0.0001) signal levels of Caco‐2 spheroids of 24.9 ± 4.3 kAU. In addition, identification of time‐dependent metabolic activity variations was also achieved. For example, after incubation for 12 d on‐chip, calcein intensity changes were only significantly elevated in NHDF spheroids, while constant fluorescent values were monitored for epithelial cell lines. These results correlate to reported variations in calcein‐AM and consequently intracellular esterase activities for different cell lines.^[^ ^38^ ^]^

Final spheroid quality evaluation involved the investigation of hypoxic conditions for a cultivation period of 12 d on‐chip. Results shown in Figure 5D reveal the presence of hypoxic conditions in all spheroids after a 12 d cultivation period. High hypoxia signals of 21.1 ± 0.9 kAU were already detected at day 3 for primary fibroblast spheroids, followed by a 235% increase in hypoxia to fluorescent intensity of 70.6 ± 1.7 kAU at day 12. The parallel increase of metabolic activity in the presence of hypoxia signals confirms the reported stimulating effect of hypoxia on dermal fibroblasts during wound healing.^[^ ^39^, ^40^, ^41^ ^]^ In contrast, epithelial tumor spheroids exhibited significantly lower hypoxic condition levels in all spheroid sizes (*P* = 0.0004) of 42.9 ± 15.7 kAU, 52.8 ± 4.5 kAU, and 16.9 ± 5.5 kAU for A549, HepG2, and Caco‐2 spheroids respectively, after 12 days postseeding. Even though cancer spheroids had the highest spheroid diameters, none of the investigated models showed hypoxia on day three, indicating higher hypoxia resilience than primary fibroblasts. Considering that healthy lung alveoli face approximately 100–110 mmHg of pO_2_ in contrast to a healthy colon, which is normoxic below ten mmHg pO_2_,^[^ ^42^ ^]^ these differences in metabolism and susceptibility toward hypoxia are not surprising and described as a response of cell models to in vitro culture conditions. Summarizing these results, we demonstrated that our microfluidic spheroid array system is capable of performing multiparametric prescreenings of critical spheroid parameters (as morphology, metabolic activity, and hypoxia) that are ultimately revealing cell type‐, spheroid size‐, and time‐specific differences.

### 2.5 Spheroid Size‐Dependent Tissue Diffusivity and Toxicity of Anticancer Drugs

**Figure 6:**
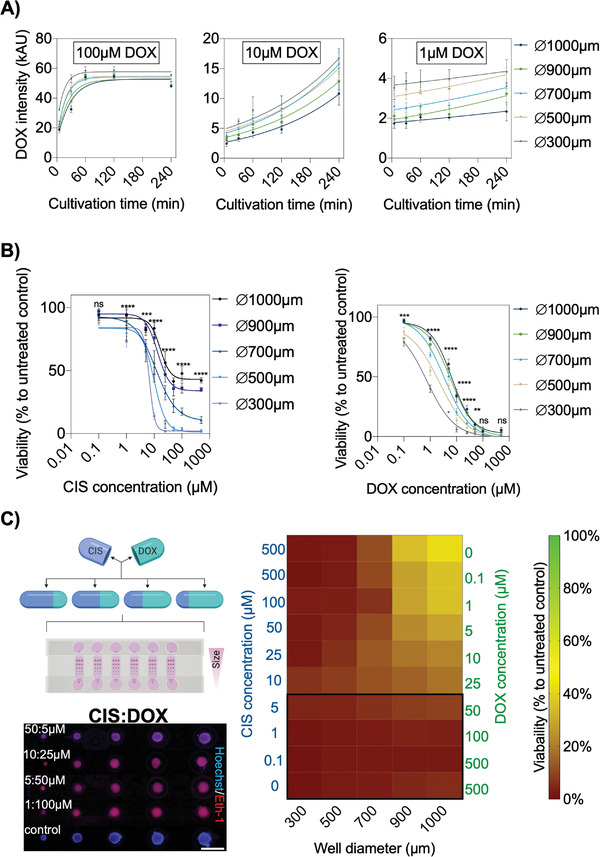
A) Monitoring of on‐chip A549 spheroid penetration of 100 × 10^−6^ m, 10 × 10^−6^ m, and 1 × 10^−6^ m doxorubicin (DOX) over a cultivation period of 4 h, *n* = 6 ± SD. B) Dose‐response relationships of CIS and DOX treated A549 spheroids of different sizes (generated in 1000, 900, 700, 500, and 300 µm microwells) in the spheroid array chip for 24 h using a dye exclusion assay (Hoechst; cell nuclei and ethidium homodimer‐1; dead cells), *n* = 4–6 ± SD. Statistical analysis of respective CIS and DOX concentrations was performed using the mixed‐effects model. (*p < 0.0332, **p < 0.0021, ***p < 0.0002, ****p < 0.0001). C) Combinatorial on‐chip drug screening of CIS and DOX in correlation to untreated controls after 24 h exposure of A549 spheroids of various dimensions, *n* = 3–6 ± SD. Corresponding fluorescent micrographs of treated different‐sized A549 spheroids of CIS:DOX for 24 h to screen drug toxicity by staining cell nuclei (Hoechst; blue) and dead cells (Ethidium homodimer‐1; red). Scale bar, 1 mm.

In the next set of experiments, the effects of anticancer drug treatment scenarios on increasing spheroid sizes were evaluated to assess toxicity shifts resulting from diffusion‐limited drug penetration. As a practical example, doxorubicin (DOX), a well‐known anticancer drug (e.g., lung and ovarian cancers), was employed to assess the ability of the microfluidic multisize spheroid array to study size‐dependent drug resistance of growing solid tumors. Initially, multisized lung cancer spheroids (A549 cell line) were treated over 4 h with the autofluorescent drug DOX to determine time‐resolved diffusion. Results shown in **Figure** **6**A (see also images in Figure S7, Supporting Information) reveal i) a Gompertzian growth at a DOX concentration of 100 × 10^−6^ m, ii) a continuous exponential growth at 10 × 10^−6^ m, and iii) a linear increase at 1 × 10^−6^ m. Additionally, significant lower signals were observed for 1 × 10^−6^ m DOX in larger spheroids, thus verifying a size‐dependent diffusion barrier resulting in increasing diffusivities over time of 2.1 ± 0.4 kAU h^−1^ in 1000 µm, 2.4 ± 0.4 kAU h^−1^ in 900 µm, 3.0 ± 0.5 kAU h^−1^ in 700 µm, 3.0 ± 0.4 kAU h^−1^ in 500 µm and 3.3 ± 0.3 kAU h^−1^ in 300 µm wells (*P* < 0.0001). Overall, diffusivity results considering all DOX concentrations and spheroid dimensions indicated an indirect proportional correlation between spheroid size and drug transport.

Since drug combinations are often used in cancer therapy, the synergistic effects of doxorubicin (DOX) and cisplatin (CIS) medications are investigated on‐chip to identify the optimal concentration ratio for, e.g., lung cancer treatment. To evaluate a potential application of the microfluidic multisize spheroid array for cancer therapy optimization studies, dose‐depended effects of CIS and DOX combinations, multisized A549 spheroids were stained with Hoechst and ethidium‐homodimer‐1 and imaged after 24 h of drug exposure. Spheroid viabilities were calculated as the ratio of cell nuclei to dead cells (see Figure S8, Supporting Information) using background‐subtracted images. Results in Figure 6B show obtained size‐dependent drug dose relationships of CIS and DOX. Interestingly, at higher CIS concentrations of 1—500 × 10^−6^ m, smaller sized‐spheroids such as 300 µm diameter displayed higher drug sensitivity than 900 µm diameter spheroids, while higher DOX concentrations resulted in similar toxicities independent of each spheroid size. Additionally, calculated Hill slopes from the sigmoidal dose–response curves suggest faster cellular responses to increasing CIS concentrations (e.g., Hill slope of ‐1.2 and ‐4.5) than DOX (e.g., Hill slope of ‐0.8 to ‐1.1). Furthermore, a size‐dependent comparison of IC_50_ values (see Figure S9, Supporting Information) between larger (e.g., 474.0. ± 64.3, 364.7 ± 41.7, 320 ± 31.2, 266.6 ± 26.7 µm) and smallest (e.g., 197.2 ± 23.1 µm in 300 µm wells) A549 lung cancer spheroids revealed that a 1.5 to 2.7‐fold higher CIS and a 2.3 to 6.9‐fold higher DOX concentration is needed to reach a 50% inhibition of spheroid viability, thus confirming the influence of spheroids size on drug response. To finally evaluate the ability of the microfluidic multi‐size spheroid array to accomplish therapy optimizations, the effect of combinatorial drug concentrations on increasing tumor sizes was investigated to identify the best CIS:DOX ratio capable of eliminating all tumor spheroids independent of their sizes. Results in Figure 6C are represented as a heat map to better visualize A540 spheroid viabilities in the presence of reciprocal CIS:DOX mixtures. Remarkably, only in the presence of 0.1–5 × 10^−6^ m CIS and 500–50 × 10^−6^ m DOX mixtures, size‐independent anticancer effects were obtained for all spheroid sizes. All other drug combinations resulted in size‐related toxicity variations, as shown in Table S1 (Supporting Information). It is important to highlight that spheroid sizes significantly impact toxicities in the presence of the pure drugs CIS and DOX even at high concentrations of 500 × 10^−6^ m, while the synergistic combinatorial effect of CIS:DOX ratio (5:50 × 10^−6^ m) effectively eliminates tumor spheroids using reduced drug concentrations (e.g., factors of 1 for CIS and 10 for DOX).

### 2.6 Spheroid Size‐Dependent Compound Penetration across an Advanced 3D Blood–Brain Barrier Model

Since compound permeability across biological barriers constitutes an important aspect in the pharmaceutical drug development process, an advanced 3D blood‐brain barrier (BBB) model was established on‐chip to monitor brain‐penetrating drugs. Although altered BBB functions are observed in several diseases of the central nervous system, little is known about possible tissue size‐dependent effects on barrier function, which could severely limit the reproducibility of current in vitro spheroid models.^[^ ^43^ ^]^ A scheme of the microfluidic spheroid triple‐culture consisting of human brain endothelial cells, pericytes, and astrocytes is shown in Figure 1C (right panel). The major advantage of the 3D model over commonly used in vitro models, including, e.g., transwells, is based on direct cell–cell contact allowing increased cell‐to‐cell interactions, which, in turn, leads to enhanced BBB integrity.^[^ ^44^ ^]^ Thus, cell numbers, ratios, and sizes may influence barrier function. To investigate the ability of the microfluidic multisized spheroid array to reliably induce the formation of BBB spheroids, human primary astrocytes (hA) and human primary pericytes (hP) were cultivated with immortalized hCMEC/D3 (human cerebral microvascular endothelial cell line D3; BEC). Initial cell density optimization was conducted using a ratio of 1:1:3 (hA:hP:BEC) to evaluate the generation of 3D BBB spheroids on‐chip under continuous perfusion for 6 d postseeding. Results in **Table** **4** show reliable production of multisize spheroids using seeding densities above 3 × 10^6^ cells mL^−1^ with optimal size‐linearity at 5 × 10^6^ cells mL^−1^ (*P* = 0.0002, *R* ^2^ = 0.9096). The results further highlight the ability of the microfluidic platform for cell culture optimization studies. To confirm the spontaneous formation and structural organization of different‐sized BBB triple‐cultures on the microfluidic spheroid array, each cell type was pre‐labeled with cell labeling fluorescent dyes to visualize human astrocytes, human pericytes, and BECs, as shown in Figure S10 (Supporting Information). As observed in previous studies,^[^ ^45^, ^46^ ^]^ astrocytes were mostly located in the spheroid core, covered by hP, and surrounded by an endothelial cell layer indicating directed self‐organization of all three cell types within differently sized spheroids.

**Table 4:** Linear regression analysis and goodness‐of‐fit (*R* ^2^) values of generated sizes of BBB spheroids after six days postseeding, including human primary astrocytes (hA), human primary pericytes (hP), and immortalized hCMEC/D3 (BEC) in a ratio of 1:1:3 in respect to initial seeding densities. Statistical significance of respective slopes was determined by analysis of covariance (ANCOVA). Data are expressed as mean value ± SD for *n* = 6. Underlined values are considered as the most optimal seeding density

	Seeding densities [cells mL^−1^]				
BBB triple‐culture	1.0 × 10^6^	2.0 × 10^6^	3.0 × 10^6^	5 × 10^6^	*p*‐value of slopes
hA:hP:BEC (1:1:3)	0.9071	0.8389	0.9096	0.9318	*P* = 0.0009

**Figure 7:**
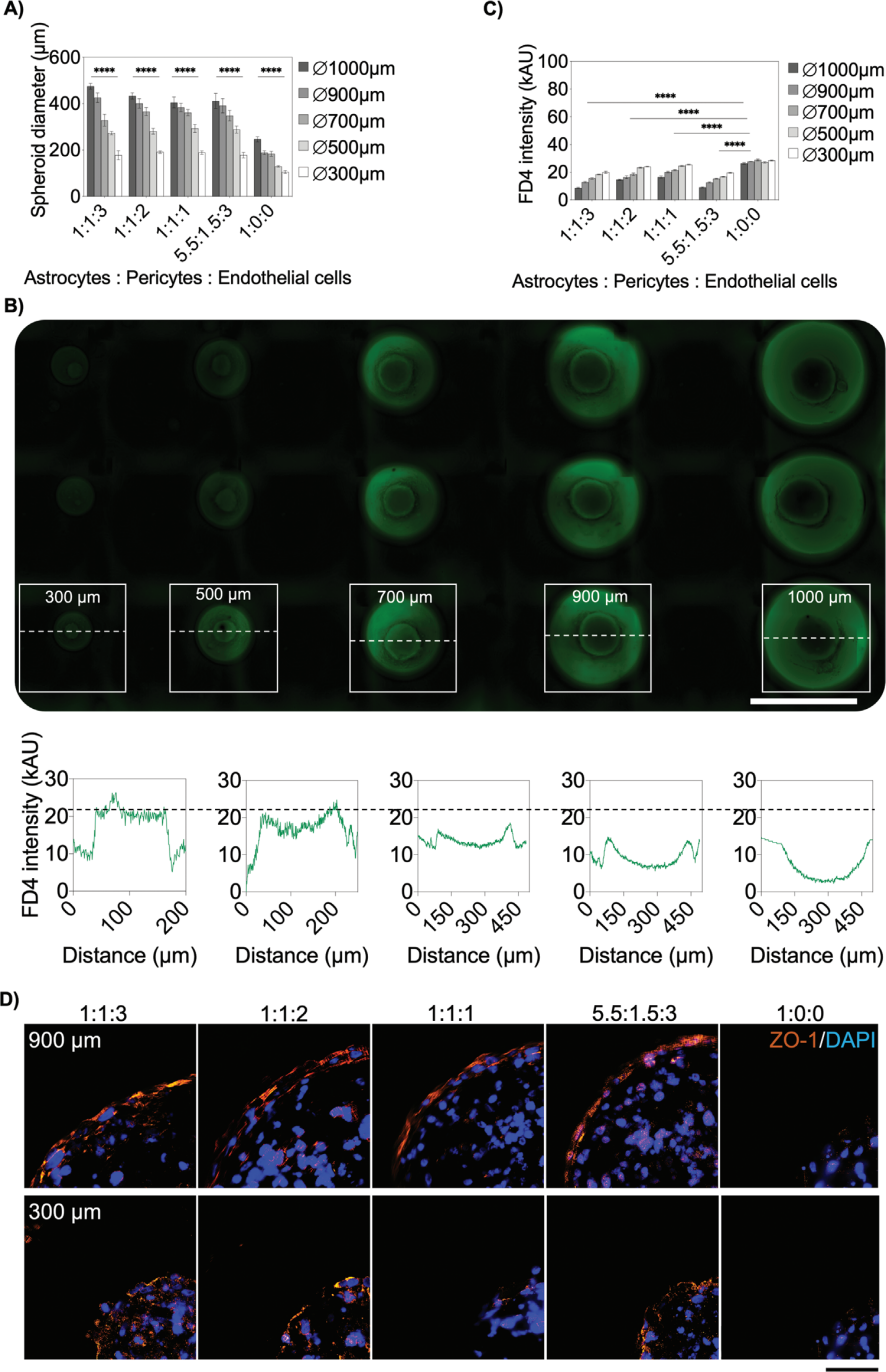
A) Spheroid diameters at the same total cell numbers and different seeding ratios of hA, hP, and hCMEC/D3 after 6 d postseeding, *n* = 6–9 ± SD. Statistical analysis was performed using the mixed‐effects model. B) Fluorescence plot profiles of 4 kDa FITC‐dextran (FD4) treated different‐sized BBB spheroids after one hour, seeded at a cell ratio of 1:1:3. Scale bar, 1 cm. C) Mean fluorescence intensities of triple‐culture spheroids of different sizes and cell ratios after 4 h of cultivation with 10 × 10^−6^ m FD4, *n* = 9–12 ± SD. Statistical analysis was performed using Dunnett's multiple comparisons test. (*p < 0.0332, **p < 0.0021, ***p < 0.0002, ****p < 0.0001). D) Immunofluorescence staining of tight‐junction associated protein ZO‐1 (orange) of large (900 µm) and small (300 µm) BBB spheroids of various cell seeding ratios. Cell nuclei were stained with DAPI (blue). Scale bar, 50 µm.

In the next set of experiments, BBB spheroids were formed with varying hA:hP:BEC cell ratios (e.g., 1:1:1, 1:1:2, 1:1:3, 5.5:1.5:3, 1:4:0, and 1:0:0) and sizes to investigate the influence of spheroid size and respective cell ratio on active and passive transport mechanisms (see also Table S2, Supporting Information). Interestingly, control experiments using astrocytes and pericytes as coculture caused single‐cell artifacts on the bottom of the microwells (see Figure S11A, Supporting Information), indicating that pericytes, by themselves, lack the ability to align on the surface of astrocytes showing their intrinsic function to mediate between brain endothelium and astrocytes.^[^ ^45^, ^47^, ^48^ ^]^ As a result of our BBB spheroid study (see **Figure** **7**A), the robust generation of a multisized spheroid triple‐culture BBB model on‐a‐chip was demonstrated since the presence of different cell ratios showed no significant size variations independent of the employed hA:hP:BEC ratio. For instance, hA:hP:BEC ratios of 1:1:3, 1:1:2, 1:1:1, and 5.5:1.5:3 (with a total seeding density of 5 × 10^6^ cells mL^−1^) resulted in the generation of 475.1 ± 38.5 µm and 177.9 ± 55.5 µm in 1000 µm and 300 µm diameter hemispherical wells. In turn, significantly smaller spheroid sizes were obtained in the presence of single‐cell type spheroids (hA:hP:BEC ratio of 1:0:0) despite similar cell seeding densities, thus indicating the impact of cell‐to‐cell interaction on spheroid growth and size. Additionally, significant differences in spheroid size‐dependent well diameter were found at all cell ratios as well as the generation of single round BBB spheroids (as shown in Figure S11B,C, Supporting Information).

In the next step, time‐resolved compound accumulation in spheroids of increasing sizes and six different cell type ratios was investigated for a period of 4 h following exposure to 10 × 10^−6^ m 4kDa FITC‐dextran (FD4). As an example of this comparative study, Figure 7B shows representative high‐resolution images of a single cell culture chamber containing 15 multisized BBB spheroids at a cell ratio of 1:1:3 (hA:hP:BECs) after 1 h of FD4 exposure (see also Figure S11D, Supporting Information). Fluorescent intensity profile analysis of spheroid cross‐sections revealed apparent size‐dependent fluorophore accumulation behavior, where FITC–dextran levels gradually decrease towards the spheroid core in the presence of larger spheroids (above 500 µm diameter wells). Results of all applied seeding ratios and sizes are shown in Figure 7C, exhibiting size‐dependent FD4 compound accumulation following a 4 h exposure. In summary, independent of spheroid size, hA monoculture spheroids showed significantly higher FD4 fluorescence intensities in a range of 29.7–31.7 kAU compared to hA:hP:BEC triple‐cultures, indicating the absence of a functional cell barrier in hA‐spheroids. Additionally, a general size‐related transport effect was revealed, suggested by a 24.6 ± 4.4% lower FD4 accumulation in large triple‐culture spheroids in 1000 µm wells than in smaller spheroids in 300 µm wells. Large BBB spheroids with a 1:1:3 cell ratio constituting the highest total number of endothelial cells (3 × 10^6^ cells mL^−1^) exhibited a distinct barrier integrity with a low FD4 signal of 19.7 ± 2.7 kAU, which was also reflected in the efficient permeability coefficient of 3.3 ± 0.6 × 10^–6^ cm s^−1^ after 1 h of FD4 incubation (see **Table** **5**). Remarkably, when shifting the ratio to 5.5:1.5:3, containing the lowest total cell numbers of pericytes (0.7 × 10^6^ cells mL^−1^) and brain endothelial cells (1.5 × 10^6^ cells mL^−1^), but the highest astrocyte fraction (2.75 × 10^6^ cells mL^−1^), results still showed low permeability of 2.9 ± 0.7 × 10^–6^ cm s^−1^, which underlined the influence of each cell type on barrier properties and revealed the importance to prescreen optimal BBB spheroid models for, e.g., compound uptake studies. Furthermore, these results are in line with reported FD4 permeability coefficients of other BBB models.^[^ ^49^, ^50^ ^]^

**Table 5:** Efficient Permeability *P*_e_ of BBB spheroids of different sizes and cell ratios after 1 and 4 h of cultivation with 10 × 10^−6^ m FD4. Data are expressed as mean value ± SD for *n* = 6

	Efficient permeability *P*_e_ (10^–6^ cm s^−1^)									
	1000 µm		900 µm		700 µm		500 µm		300 µm	
BBB seeding ratio (hA:hP:BEC)	1 h	4 h	1 h	4 h	1 h	4 h	1 h	4 h	1 h	4 h
1:1:3	3.3 ± 0.6	7.8 ± 1.3	4.5 ± 1.1	8.3 ± 1.1	4.9 ± 2.0	8.9 ± 2.3	5.2 ± 0.5	9.1 ± 1.3	6.2 ± 2.9	10.6 ± 2.3
1:1:2	6.0 ± 0.5	8.8 ± 1.4	7.5 ± 1.6	9.9 ± 1.4	8.1 ± 2.8	10.0 ± 1.8	8.9 ± 1.1	11.3 ± 2.1	9.9 ± 2.4	11.4 ± 3.0
1:1:1	7.0 ± 2.2	11.5 ± 2.1	9.2 ± 1.4	12.7 ± 1.4	9.6 ± 1.8	14.2 ± 1.4	10.4 ± 2.3	13.3 ± 2.8	11.1 ± 3.7	13.5 ± 1.2
5.5:1.5:3	2.9 ± 0.7	7.1 ± 1.5	4.4 ± 1.0	8.3 ± 1.9	5.1 ± 1.3	8.7 ± 2.2	4.6 ± 0.7	9.5 ± 2.3	5.4 ± 2.0	11.2 ± 1.1
1:0:0	11.5 ± 3.0	12.7 ± 1.9	11.8 ± 3.3	13.8 ± 3.3	12.1 ± 2.4	14.6 ± 2.9	12.4 ± 3.4	15.3 ± 2.6	14.1 ± 3.0	16.9 ± 3.6

In order to analyze barrier integrity in more detail, differences in localization and continuity at the spheroid's outer rims of tight junction‐associated protein zonula occludens‐1 (ZO‐1) were investigated by immunofluorescence staining of histological sections (see Figure 7D). Large triple‐culture spheroids generated in 900 µm hemispherical wells showed increasing ZO‐1 localization and thickness proportional to endothelial cell content. Additionally, small spheroids (300 µm) displayed weaker and more discontinued ZO‐1 signals correlating with the elevated FD4 permeability. As expected, hA spheroids with no endothelial barrier were void of ZO‐1 signal at the outermost spheroid surface with the highest FD4 permeability values. Overall, results of our microfluidic 360‐spheroid array indicate that identification and pre‐screening of barrier properties prior high‐throughput testing should be performed multiparametric since parameters as spheroid size or cell‐composition alone fail to provide conclusive evidence concerning transport properties and best performing BBB models.

**Figure 8:**
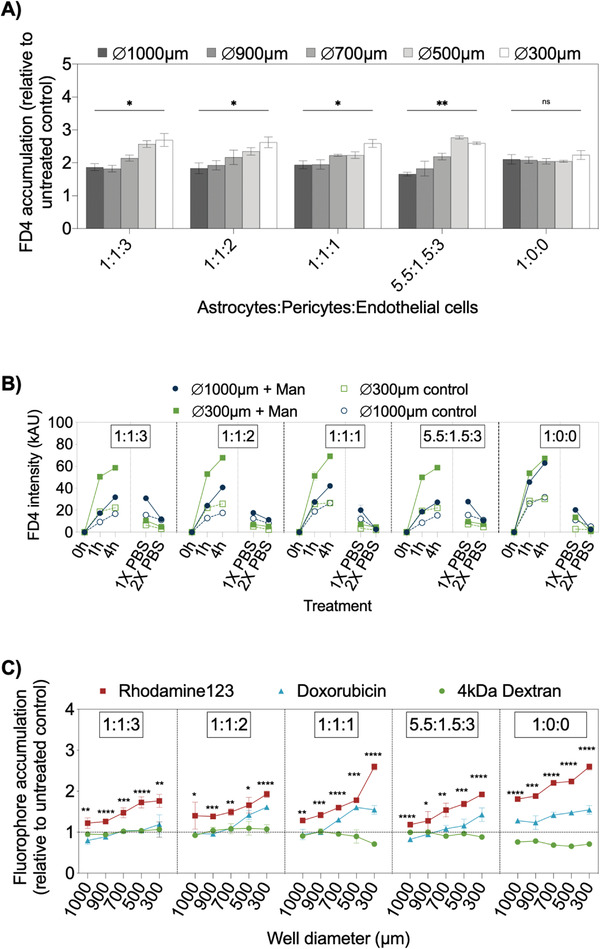
A) Accumulation fold change of 4 kDa‐FITC dextran (FD4) of mannitol treated BBB spheroids at various cell ratios and sizes in correlation to untreated controls after 4 h of incubation. Statistical analysis was performed using one‐way ANOVA, *n* = 3 ± SD (*p < 0.0332, **p < 0.0021, ***p < 0.0002, ****p < 0.0001). B) Time‐resolved FD4 intensity profiles of largest (1000 µm) and smallest (300 µm) BBB spheroids during incubation of treated (+1.6 m mannitol) and untreated BBB spheroids, *n* = 3 ± SD. C) Effects of 100 × 10^−6^ m P‐gp inhibitor verapamil on fluorophore accumulation of 10 × 10^−6^ m 4 kDa FITC–dextran, 10 × 10^−6^ m rhodamine123, 1 × 10^−6^ m doxorubicin after 1 h of incubation in BBB spheroids in correlation to untreated control without verapamil, *n* = 3 ± SD. Statistical analysis of significance between fluorophore accumulation at each spheroid size was performed by one‐way ANOVA (*p < 0.0332, **p < 0.0021, ***p < 0.0002, ****p < 0.0001).

Final practical evaluation of the microfluidic multisize spheroid array involved the investigation of spheroid size‐related effects on FD4 accumulation in the presence of the BBB opening agent mannitol, which has been exploited as a drug and therapeutic agent delivery system for facilitating the entrance of therapeutic biologics into the brain.^[^ ^51^ ^]^ Results of FD4 accumulation studies are shown in **Figure** **8**A where a clinically relevant mannitol concentration (e.g., 1.6 m),^[^ ^52^, ^53^ ^]^ was applied as an indicator of barrier integrity loss in our BBB‐chip model. For instance, a comparable FD4 signal increase of 1.6 to 2.8‐fold was observed in all spheroid samples independent of the used cell ratios after a four‐hour incubation period, thus confirming the barrier opening effect of mannitol. However, mannitol treatment of triple‐culture spheroids resulted in an indirect proportional increase of FD4 accumulation with decreasing spheroid size. In more detail, a size‐dependent variation in FD4 accumulation exhibiting an increasing mean FD4 signal fold change relative to untreated control of 1.8 ± 0.1, 1.9 ± 0.1, 2.2 ± 0.1, 2.5 ± 0.2, and 2.6 ± 0.1 was observable in smaller BBB spheroids sizes. Additional time‐resolved FD4 accumulation tests using largest (1000 µm wells) and smallest BBB spheroids (300 µm wells) were conducted to assess passive uptake kinetics of untreated and mannitol‐treated spheroids of varying cell ratios as shown in Figure 8B. Generally, smaller spheroids revealed 79.8 ± 19.8% significantly higher FD4 signal intensities in mannitol‐treated triple‐culture spheroids than larger ones (e.g., 46.6%) and untreated spheroids of similar sizes (31.2%). In contrast, astrocyte monoculture spheroids showed comparable fluorescence signals in both sizes during mannitol treatment, confirming the absence of BBB endothelium. Furthermore, results of treated spheroids showed similar FD4 transport kinetics in accumulation of 16.3 ± 4.6% and 17.8 ± 4.0% with brain endothelial (hA:hP:BEC) ratios of 1:1:3 and 5.5:1.5:3, while increased uptake levels of 28.1 ± 0.8%, 29.6 ± 9.4%, and 25.2 ± 2.8% were observed in spheroids consisting of smaller BEC fractions of 1:1:2, 1:1:1, and 1:0:0, respectively. Similar phenomena could also be observed after washing spheroids with PBS (w/wo mannitol) to monitor FD4 efflux. For instance, after 1× PBS, FD4 signals in larger spheroids remained stable in treated and untreated spheroids at 1:1:3 and 5.5:1.5:3 ratios, in contrast, mannitol treated spheroids of 1:1:2, 1:1:1, and 1:0:0 fractions showed significant signal reductions relative to control of 48.1 ± 3.4%, 50.8 ± 4.1, and 33.2 ± 9.0%, respectively. Interestingly, smaller spheroids revealed a higher signal decrease for all cell ratios in the presence of mannitol versus untreated control of approximately 74%. Overall, these paracellular tightness studies not only verify the barrier opening effect of mannitol in triple‐culture BBB spheroids based on astrocytes, pericytes, and brain endothelial cells but also highlight a spheroid size‐related modulation on passive, paracellular transport properties.

In the last set of experiments, active transport of compounds into our advanced 3D BBB model was investigated. Here, spheroids were treated with the potent P‐gp inhibitor verapamil, which is known to inhibit efflux of the P‐gp substrates rhodamine123 and doxorubicin,^[^ ^54^, ^55^ ^]^ to examine efflux pump activities of multisized BBB spheroids. Results in Figure 8E show compound accumulation by measuring fluorescence intensities found in the spheroid cores of increasing sizes and different cell ratios as an indicator of active efflux of rhodamine123 (RHO), doxorubicin (DOX), and 4kDa FITC‐dextran (FD4) in the absence and presence of verapamil. An effective inhibition of the efflux pump activities in verapamil‐treated multisized spheroids was found independent of the cell ratios, resulting in an increased accumulation compared to FD4 of 31.3%–43.8% of the P‐gp substrates RHO and 8.6%‐24.9% in DOX‐treated multisized, triple‐culture BBB spheroids. In contrast, monoculture spheroids indicated enhanced spheroid core accumulation, shown by a 66.1% ± 6.5% increase of RHO and 47.4 ± 8.8% of DOX in correlation to FD4. These results showed that verapamil did not affect FD4 uptake, verifying an effective blockade of the ABC transporter and enabling an increased transcellular accumulation of RHO and DOX. In turn, DOX accumulation was mainly unaffected by verapamil treatment in the largest triple‐culture spheroids, while little increase of fluorescence signals was found in smaller spheroids. This underlines that a spheroid size‐dependent active compound accumulation was observed with clear differences in RHO, DOX, and FD4 uptake rates in the largest triple‐culture spheroids (1000 µm wells) of *P* = 0.0006 in comparison to smallest spheroids (300 µm wells). These findings strongly suggest that RHO accumulation in triple‐culture spheroids was enhanced at all seeding ratios and spheroid sizes due to treatment with verapamil, while DOX accumulation was mainly observed in small spheroids, thus highlighting the importance of uniform spheroid dimensions and cellular ratios for BBB compound uptake studies. Therefore, the perfused BBB spheroid chip model represents a scalable cell culture tool due to the simplicity of the approach to establish 3D aggregates and the capability to screen multiple BBB spheroid architectures for studying drug transport mechanisms on a single device.

## 3 Conclusion

Current 3D spheroid methodologies generate spheroids that vary in size, morphology, and complexity. This leads to challenges in obtaining standards concerning culture and assay protocols as well as output data for any given cell type and tissue model.^[^ ^17^ ^]^ Next‐generation spheroid technologies, therefore, need to ensure higher reproducibility, multiparametric analysis, compatibility of readout techniques, and better automation, to establish standardized and validated in vitro 3D tissue models with improved quality, consistency, and predictive capacity. To address these shortcomings, we designed, fabricated, and tested a microfluidic chip system capable of reliably generating a large number of spheroids of defined sizes, which can be readily integrated into pharmaceutical workflows. Overall, the device is easy to operate, robust, and potentially compatible with other technologies, such as robotic pipetting, live‐cell imaging, plate readers, and laboratory tilting platforms.

To date, the majority of commercial and academic approaches for spheroid generation are still based on static culturing conditions such as ultra‐low attachment plates (e.g., Aggrewell plates) or 384‐hanging drop systems. Recent studies have shown that microfluidic technology has contributed significantly to spheroid research by addressing the deficiencies of static methods such as variable spheroid diameters, laborious handling, high reagent consumption, and better recapitulation of the in vivo microenvironment. Even though perfused spheroid culture platforms have been developed over the last decade,^[^ ^36^ ^]^ these microfluidic devices have not entered the market yet. This can be attributed to extensive operational know‐how requirements, the lack of scale‐up and parallelization possibilities as well as limited throughput of the devices (e.g., 24 or 96 spheroids on one plate).^[^ ^25^, ^32^ ^]^ Here, our plug‐and‐play microfluidic multispheroid array in well‐plate format shows great potential to enable user‐friendly, medium‐to‐high‐throughput microfluidic prescreening (e.g., culture establishment and optimization) and screening applications (e.g., anticancer drug testing) of up to 360 spheroids. The presented system also allows the stacking of multiple plates on a laboratory rocker platform, permitting an easy scale‐up of spheroid production and cultivation on‐chip. This extent of parallelization constitutes a major improvement of throughput compared to currently reported microfluidic spheroid systems and is competitive to other mid‐to‐high‐throughput plates. Another feature of perfused systems is that spheroids can be cultured in a dynamic micromilieu to better recapitulate the native tissue environment by controlling continuous flow and shear stress, improving spheroid function, and long‐term cultivation performance.^[^ ^56^, ^57^, ^58^, ^59^ ^]^ A significant advantage includes the parallel production of 15 spheroids of five different sizes by applying only one cell density in a single pipetting step in contrast to numerous dilution series and laborious pipetting procedures that are necessary for traditional well‐plate cultures. Consequently, the presented approach enables direct monitoring of spheroid size effects under various treatment scenarios on a single device, with minimum user manipulation avoiding needless pipetting errors and excessive demand of expensive culture media or test reagents. Furthermore, the presented microfluidic technology facilitates a direct automated and reproducible control of spheroid size and morphology under continuous perfusion with relative standard deviations of 12% or less in a diameter range between 90 µm and 900 µm. Nonetheless, a current drawback of our novel microfluidic spheroid array is that manual delamination of the reversible sealing and pooling of spheroids for proteomic/genomic end‐point detection is still necessary. Since this time‐consuming step is not feasible for automation, future design considerations include one‐channel/one‐size and one‐chip/one‐size strategies, which allow single‐step harvesting/pooling procedures for more in‐depth functional analysis of a uniform‐sized sample population. Subsequently, any combination of four microfluidic spheroid array inserts can be used on a microplate‐format and adapted to the specific research question and analytical requirements.

Overall, the results of our multisized spheroid study verified an apparent size‐dependent effect of compound penetration, toxicity, and uptake. Chemotherapeutic drug transport and its uptake by tumor cells are strongly dependent on solid tumor properties, especially size, representing a crucial parameter for drug sensitivity.^[^ ^60^, ^61^, ^62^ ^]^ Here, we demonstrated size‐dependent transport kinetics of the fluorescent drug doxorubicin, known for its high efficacy in lung and ovarian cancers,^[^ ^63^, ^64^, ^65^ ^]^ using A549 lung cancer spheroids. The impact of spheroid size was demonstrated by significant IC_50_ differences up to 160% in a single treatment regime in A549 lung cancer spheroids. Next, the synergistic therapeutic effect of cisplatin,^[^ ^66^, ^67^ ^]^ a DNA synthesis inhibitor, and doxorubicin,^[^ ^68^, ^69^ ^]^ known to inhibit the topoisomerase II (TOP2) pathway, was demonstrated by identifying the ideal ratio and minimum concentrations needed to overwhelm the cellular repair mechanisms in tumor spheroids. This combinatorial therapy aspect is particularly critical,^[^ ^70^, ^71^, ^72^ ^]^ since both DOX and CIS exhibited severe side effects and drug resistance in clinic.^[^ ^73^ ^]^ As a final practical example, compound permeability in BBB spheroids was screened to study BBB spheroid size effects on barrier function.^[^ ^43^ ^]^ During the past years, different 3D BBB models and approaches have been established to mimic the BBB's biological niche by assembling spheroids with a range of distinctive ratios of hAs, hPs, and BECs into a BBB−like model.^[^ ^45^, ^46^, ^48^ ^]^ For the first time, successful integration of triple‐culture BBB spheroids into a microfluidic setup as well as a parallel screening of BBB spheroid size effects on self‐organization and compound transport was demonstrated, representing a novel approach for future experimental design strategy optimizations. Initially, the influence of seeding densities and cell ratios on BBB spheroid sizes and compound diffusivity was evaluated in our work to establish an improved and reliable BBB model. Additionally, the penetration enhancer mannitol, which is applied in, e.g., glioblastoma patients inducing the opening of endothelial tight junctions to allow the passage of chemotherapeutics that normally cannot enter the parenchyma,^[^ ^74^, ^75^ ^]^ was used to demonstrate spheroid size‐dependent paracellular transport kinetics. The results indicated significant differences in larger and smaller spheroids independent of the employed cell ratios during mannitol treatment. The inhibition of the P‐gp efflux pump using verapamil further revealed increased accumulation of RHO and DOX in our BBB model with the exemption of smaller spheroids, where increased DOX accumulation was recorded, thus highlighting the importance of size for the optimization of BBB properties.

In conclusion, we demonstrated the compatibility, usability, and throughput of a microfluidic platform to produce and measure complex multisized spheroids, accelerating optimization and screening protocols of an advanced in vitro model and ultimately increase predictive accuracy in basic and preclinical biomedical research.

## 4 Experimental Section

##### Microfluidic Multisize Spheroid Array Fabrication

The microfluidic spheroid array chip was fabricated by double‐casting of polydimethyl siloxane (PDMS). The master mold, including microwells and channel structures, was manufactured in polymethyl methacrylate (PMMA) by CNC micromilling (Denz‐Biomedical, Austria). PDMS (Sylgard 184 Silicon Elastomer, Farnell, Austria) was mixed with the curing agent in a weight ratio of 10:1. The polymer was degassed in a vacuum chamber for 1 h, poured onto the PMMA structure, and baked for 2 h at 80 °C. The structure was peeled off from the PMMA matrix subsequently and was hard baked for 48 h at 90 °C. As a result, the final PDMS mold for biochip channel structure fabrication was obtained. To remove PDMS chip structures from molds properly, the surface of the PDMS mold was plasma‐activated and silanized with trichloro(1H,1H,2H,2H‐perfluorooctyl) silane (Sigma‐Aldrich, Austria) for 10 min under vacuum and baked for 1 h at 80 °C. Molds for top layer, including reservoirs, were 3D printed by iMaterialise (Denmark). PDMS master mix was poured into 3D printed molds and baked for 2 h at 70 °C. Before bonding, each channel was coated with 0.5% wt antifouling Lipidure‐CM5206 solution (AMSbio, UK) for 1 h at 80 °C. Holes of 1.5 mm diameter were punched through the reservoir layer with biopsy punchers to generate perfusion connectors between the reservoirs and the channels. The two PDMS layers (channel structure layer and top layer with reservoirs) were bonded by O_2_‐plasma activation for 30 s, 0.9 mbar, 200 W (Diener, Germany) and baked at 80 °C overnight.

##### Cell Culture Handling and Cultivation Procedures

Caco‐2 (HTB‐37, ATCC, USA), and normal human dermal fibroblasts (NHDF; CRL‐2522, ATCC, USA) were cultured with Dulbecco's minimal essential medium (DMEM; Sigma‐Aldrich, Austria) supplemented with 10% fetal bovine serum (FBS; Sigma‐Aldrich, Austria) and 1% antibiotic/antimycotic solution (Sigma‐Aldrich, Austria). HepG2 cells (HB‐8065, ATCC, USA) were cultivated with supplemented minimal essential medium (MEM; Sigma‐Aldrich, Austria) with 10% FBS and 1% antibiotic/antimycotic solution (Sigma‐Aldrich, Austria), and A549 cells (CCL‐185, ATCC, USA) were cultivated in Hams F12K Medium (Sigma‐Aldrich, Austria) with 10% FBS and 1% antibiotic/antimycotic solution (Sigma‐Aldrich, Austria). All cell types were cultivated in T75 cell culture flasks at 37 °C in 5% CO_2_ humidified atmosphere as adherent monolayers. Cells were washed with 1× phosphate‐saline buffer (PBS; Sigma‐Aldrich) at a confluency of 70–80%, and 0.5% trypsin–EDTA (Sigma‐Aldrich, Austria) was added for 10 min to detach cells. After detachment, respective growth medium was added, and cells were centrifuged at 140 g for 5 min. Medium was removed, and the cell pellet was diluted to required cell densities. For blood‐brain barrier experiments, human primary astrocytes (hA; SC‐1800‐5, Provitro AG, Germany) were cultured in astrocyte medium AM (ScienCell, USA) supplemented with 2% FBS (ScienCell, USA), 1% of penicillin/streptomycin (ScienCell, USA), and 1% astrocyte growth supplement (ScienCell, USA). Human primary pericytes (hP; SC‐1200, Provitro AG, Germany) were cultivated in pericyte medium PM (ScienCell, USA) supplemented with 2% FBS (ScienCell, USA), 1% of penicillin/streptomycin (ScienCell, USA), and 1% pericyte growth supplement (ScienCell, USA). Human primary astrocytes and human primary pericytes were cultured on 10 µg mL^−1^ poly‐l‐lysine (ScienCell, USA) coated culture flasks. Human cerebral microvascular endothelial cells (hCMEC/D3; SCC066, Merck Millipore, Germany) were cultured on 0.5% gelatin‐coated culture flasks (SERVA Electrophoresis GmbH, Germany) in EBM‐2 (Lonza, Swiss) containing 5% FBS (Sigma‐Aldrich, USA), 1% penicillin/streptomycin (Biochrom GmbH, Germany;) as well as 10 × 10^−3^ m HEPES (Sigma‐Aldrich, USA), 5 µg mL^−1^ ascorbic acid (Sigma‐Aldrich, USA) and 1 ng mL^−1^ hbFGF (Sigma‐Aldrich, USA). For experimental use, astrocytes were maintained between passages 3 and 8, pericytes between passages 4 and 8, hCMEC/D3 cells between passage 21 and 32. Cells were cultivated as previously described.^[^ ^76^ ^]^

To visualize the location of each cell type in BBB triple‐culture spheroids, hA were labeled with CellTracker Deep‐red Dye (5 × 10^−6^ m; Thermo Fisher Scientific, USA), hP with NucBlue Live Cell Stain (1 drop; Life Technologies, USA) and hCMEC/D3 were labeled with CellTracker Orange CMDA dye (5 × 10^−6^ m; Thermo Fisher Scientific, USA). After cell detachment, cells were centrifuged at 300 g for 5 min and washed with DMEM without further supplements (Gibco, Thermo Fisher Scientific, USA). Cells were mixed with each dye in DMEM without further supplements (Gibco, Thermo Fisher Scientific, USA) and incubated for 30 min at 37 °C in the water bath. After centrifugation, cells were resuspended in the corresponding culture medium and the BBB spheroid formation protocol was continued.

##### Chip Loading and Cell Seeding Protocol

Before cell seeding, chips were filled with 70% ethanol and placed in an ultrasonic bath to remove air bubbles. Chips were sterilized by washing 3× with 200 µL of 70% ethanol and three times with 200 µL of 1× PBS (Sigma‐Aldrich, Austria) supplemented with 1% of penicillin/streptomycin (Sigma‐Aldrich, Austria) to clear the channel from ethanol. Chips were maintained and incubated in quadriPERM chambers (Sarstedt, Austria) filled with 2 mL 1× PBS supplemented with 1% antibiotic/antimycotic solution (Sigma‐Aldrich, Austria) to avoid liquid evaporation. Prior to cell seeding, PBS was removed from all reservoirs, and preconditioned with 200 µL cell culture medium. After removal of medium, 100 µL of a cell suspension was added to each channel. The quadriPERM with the chips was placed on the rocker platform and set to a flow rate of 4 µL min^−1^ at 1° tilting angle and 1 rpm. On the next day, channels were flushed with 200 µL growth medium to remove excess cells. Growth medium in the chips was changed every 2 d.

##### CFD Simulation

CFD (computational fluid dynamics) modeling was carried out using Ansys Fluent 6.3.26, (www.ansys.com), a general‐purpose finite volume CFD solver. The computational mesh for the 3D fluid flow problem consisted of about 120 000 hexahedral control volumes. Next, steady‐state snapshots representative for the physical movement were identified and the flow geometry was oriented accordingly. Wall boundaries were treated as ideally smooth and no‐slip (zero flow velocity at the wall), inlet and outlet were set to pressure boundary conditions (reference pressure *p* = 1 atm/101325 Pa at the lower fluid column). Gravity or equivalent pressure of a virtual water column was used as single fluid phase (Newtonian fluid, constant dynamic viscosity) and the flow was idealized as isothermal (reference temperature *T* = 25 °C) and incompressible (constant density). Second or higher‐order discretization schemes were selected for continuity equation (mass conservation) and Navier‐Stokes equations (momentum conservation). Due to the small geometrical features and the low fluid velocities, the flow can safely be considered as laminar (*R*e << 1). Simulations were carried out on the cluster server cae.zserv.tuwien.ac.at (operated by the IT department of TU Wien, www.zid.tuwien.ac.at). Base on the steady‐state snapshot CFD results, correlations were derived to set up a fast 1D mass balancing tool to calculate and analyze the transient flow behavior inside the spheroid chamber.

##### Flow Rate Measurements

Measuring the flow rate in the chip at various tilting angles was achieved by setting an assembled platform (Rocker Platform Shaker 444‐0756, VWR, Austria) on a tilting stage at a defined angle *α*. Medium reservoirs were filled with stained cell culture medium to ensure steady flow from the experiment's beginning. To calculate the volumetric flow rate, measurements of the angle associated change of liquid column height were made. Images from the neutral setting and the maximum angle were taken and change in liquid column height Δ*h* was measured with ImageJ FIJI (NIH, USA), and ΔP was calculated using the tilting‐dependent hydrostatic pressure difference in Equation (1):

(1) ΔP=ρgΔh

and the hydrodynamic resistance *R*_h_ as shown in Equation (2) which is the sum of the hydraulic resistance of the microfluidic tissue culture channel (*R*_r_) and both tubular connecting channels (*R*_t_; Equations (3) and (4))

(2) $$R_{h}=R_{r}+2R_{t}\;$$

(3) $$R_{r}=\frac{12\eta l}{wh^{3}}\;\left[1-\frac{192h}{\pi^{5}w}\tan h{\left(\frac{\pi w}{2h}\right)}^{-1}\right]\;$$

(4) $$R_{t}=\frac{8\eta l}{\pi r^{4}}\;$$

The hydrodynamic resistance is given by the dimensions of the culture channel and the fluid properties, where *l* is the length, *w* is the width, *h* is the height, and *η* is the dynamic viscosity. The volumetric flow rates *Q* through the device is proportional to Δ*P* for a given channel hydraulic resistance* Rh* which is described in Equation (4):

(5) $$Q=\Delta PR_{h\;}$$

##### Evaluation of Spheroid Esterase Activity and Hypoxia

The calcein‐AM (Invitrogen, Austria) solution was prepared in growth medium for each cell type with a concentration of 0.5 µL stock per mL growth medium. Growth medium was removed from reservoirs, and 200 µL of the calcein‐AM solution was added to each reservoir. After incubation of 30 min under standard cell culture conditions, the spheroids were imaged. All further calcein determinations were performed according to the same protocol with corresponding growth medium. To monitor hypoxia on‐chip, 10 × 10^−6^ m of Image‐iT Red Hypoxia Reagent (Invitrogen, Austria) was prepared in respective cell growth medium. As shown in Figure S6 (Supporting Information), culture medium was gently removed from reservoirs, and 200 µL of the 10 × 10^−6^ m hypoxia reagent was applied. The chip was incubated for 1 h at 37 °C and 5% CO_2_ in a live‐cell incubator (Pecon, Germany) and imaged using TRITC filter (ex 530 nm, em 645 nm) by IX83 live‐cell microscope (Olympus, Germany).

##### Doxorubicin Penetration Study

After 3 d of cultivation, medium was removed from chip reservoirs and 200 µL of fresh medium supplemented with 100 × 10^−6^ m, 10 × 10^−6^ m and 1 × 10^−6^ m of doxorubicin (Sigma‐Aldrich, Germany) was added, followed by the incubation at 37 °C and 5% CO_2_ in a life cell incubator (Pecon, Germany) where real‐time tracking of fluorescence intensities was performed. Images were taken after 5, 60, 120, and 460 min using a FITC filter (ex 485, em 530; IX83, Olympus, Germany).

##### Anticancer Drug Screening

A549 cells were seeded at a concentration of 1 × 10^6^ cells per mL and cultivated for 3 d under standard cell culture conditions under bi‐directional flow. Stock solutions of 10 × 10^−3^ m cisplatin (Sigma‐Aldrich, Austria) in DMSO and 10 × 10^−3^ m doxorubicin (Sigma‐Aldrich, Austria) in PBS were prepared. Doxorubicin and cisplatin were dissolved in cell culture medium to yield concentrations of 0.5 × 10^−6^, 1 × 10^−6^, 5 × 10^−6^, 10 × 10^−6^, 25 × 10^−6^, 50 × 10^−6^, and 500 × 10^−6^ m for the treatment of A549 spheroids. Cell culture medium within the channels of the devices was replaced with drug containing medium and incubated for 24 h prior to cell death analyses. One channel on a separate device was used as an untreated control. Following the incubation, drug solutions were removed, and 10 µg mL^−1^ Hoechst 33342 (Invitrogen, Austria) and 4 × 10^−6^ m ethidium–Homodimer 1 (Invitrogen, Austria) applied. After incubation for 30 min, spheroids were imaged using DAPI (ex 390 nm, em 460 nm) and TRITC filters (ex 530 nm, em 645 nm). Raw fluorescence signals were processed as described in 2.11, and dose–response curves were generated by Sigmoidal‐4PL nonlinear regression analysis.

##### Evaluation of BBB Permeability

Multicellular BBB spheroids were formed through a two‐step cell seeding protocol. First, human primary astrocytes were seeded on‐chip by injecting 100 µL of cell suspension into the channels and incubated overnight at 37 °C, 5% CO_2_ in a cell culture incubator to allow the assembly of astrocyte spheroids. Second, human primary pericytes and hCMEC/D3 were mixed at defined ratios, and 100 µL of the cell suspension were injected into respective microchannels to form multilayered BBB spheroids and incubated for 6 d. Cells were seeded at 1:1:3, 1:1:2, 1:1:1, 5.5:1.5:3, 1:4:0, and 1:0:0 rations of astrocytes:pericytes:hCMEC/D3 at total seeding densities of 5 × 10, ^6^ 3 × 10^6^, 2 × 10^6^ and 1 × 10^6^ cells mL^−1^. Medium was changed every 2 d with respective medium ratios for each cell type. For real‐time permeability experiments, spheroids of different cell type rations were generated at a seeding density of 5 × 10^6^ cells mL^−1^. Spheroids were incubated with 200 µL of the paracellular marker fluorescein isothiocyanate‐dextran (10 × 10^−6^ m; FD4; 4 kDa; Sigma‐Aldrich, USA, 1 × 10^−3^ m stock solution of FD4 ultrafiltered with Amicon tubes with a cutoff 3 kDa to separate from residual, free FITC) in the supplemented EBM‐2 medium for 4 h at 37 °C with 5% CO_2_ in live‐cell incubator (Pecon, Germany) and imaged after one and 4 h. For mannitol experiments, BBB spheroids were treated with 200 µL of 1.6 m D‐Mannitol (Fluka, Austria) and 10 × 10^−6^ m FD4 in supplemented EBM‐2 medium for 4 h and washed two times with 200 µL of 1× PBS containing 1.6 m d‐Mannitol. To monitor P‐gp activity, the BBB‐spheroids were pretreated with 100 × 10^−6^ m of the P‐gp blocker verapamil (Sigma‐Aldrich, Austria) in serum‐free EBM‐2 for 15 min and exposed to a mixture of 100 × 10^−6^ m verapamil and 10 × 10^−6^ m rhodamine123 (Sigma‐Aldrich, Austria), 100 × 10^−6^ m verapamil and 1 × 10^−6^ m doxorubicin (Sigma‐Aldrich, Austria) or 100 × 10^−6^ m verapamil and 10 × 10^−6^ m FD4 for 1 h. Spheroids were treated with rhodamine123, doxorubicin, or FD4 with 0.16% DMSO (PanReac AppliChem, Austria) as control on separate chips.

To calculate the efficient permeability *P*_e_ of BBB spheroids, Equation (5) was used as described elsewhere:^[^ ^77^ ^]^

(6) $$P_{e}=\frac{-\ln\left(1-\frac{C_{s}}{C_{\mathrm{equilibrium}}}\right)}{A_{s}\left(\frac{1}{V_{m}}+\frac{1}{V_{s}}\right)t}\;$$

where *C*_s_ is the FD4 intensity in the spheroid at time *t*, *A*_s_ is the surface area of the spheroid, *V*_m_ is the volume of medium, *V*_s_ is the volume of spheroid, and *t* is the incubation time. *C*_equilibrium_ is obtained by the Equation (6):

(7) $$C_{\mathrm{equilibrium}}=\frac{C_{m}V_{m}+C_{s}V_{s}}{V_{m}+V_{s}}\;$$

where *C*_m_ is the FD4 intensity in the medium at time *t*.

##### Immunohistochemistry

After 6 d of culture on‐chip, BBB spheroids were washed twice with 1× PBS (Sigma‐Aldrich, Austria) and fixed with 4% paraformaldehyde in 1× PBS (containing Mg^2+^ and Ca^2+^; Sigma‐Aldrich, Austria) at 4 °C overnight. Individual BBB spheroids were harvested by cutting off the PDMS chip's top layer with a scalpel and transferred to Eppendorf tubes. The spheroids were embedded in paraffin and sliced into 4 µm serial sections, deparaffinized in xylene, and rehydrated in a graded alcohol series. Antigen retrieval was performed by keeping rehydrated sections in a 10 × 10^−3^ m sodium citrate buffer pH 6.0 (Sigma‐Aldrich, Austria) for 20 mi at 100 °C in a steamer. Blocking was conducted by exposure to 10% goat serum with 1% BSA in 1× Tris‐buffered saline pH 7.6 (TBS; Sigma‐Aldrich, Austria) for 2 h at room temperature. Samples were incubated with polyclonal rabbit anti‐ZO‐1 (1:100; 21773‐1‐AP, ProteinTech, Germany) at 4 °C overnight. After washing with 1× TBS, secondary Alexa Fluor 555 goat antirabbit IgG (1:1000; A32732, Invitrogen, Austria) was applied for 1 h at room temperature. Nuclei were counterstained with DAPI (1:1000; Thermo‐Fischer, Austria). Images were acquired by Olympus IX83 live‐cell microscope using DAPI (Ex: 350/Em: 470) and TRITC filters (ex 530 nm, em 645 nm). Images of fluorescent immunohistochemical staining were taken using equal filter and acquisition parameters to assure comparable conditions.

##### Image Acquisition and Data Processing

To investigate spheroid size and morphology, bright‐field images were taken using an IX83 microscope (Olympus, Austria) equipped with temperature, CO_2_, and O_2_ control (Peacon, Germany) and high‐resolution camera (Hamamatsu, Germany). For imaging of the whole cultivation channel, MIA scans were conducted using 4× and 10× magnification. All images were processed by ImageJ (NIH, USA). For morphometric analysis, micrographs were converted to 8‐bit, threshold was adjusted, and area, perimeter, roundness, and solidity were measured by the function of Analyze Particles. Roundness was calculated as described in Equation (7):

(8) $$\mathrm{Roundness}\;=\frac{4A_{s}}{\pi a^{2}}\;$$

where is the *A*_s_ is the spheroid surface area and *a* is the major axis of the diameter. Spheroid solidity was defined using Equation (8):

(9) $$\mathrm{Solidity}\;=\frac{A_{s}}{A_{c}}\;$$

where *A*_c_ is the convex area.

Center‐to‐center distances were determined mathematically by calculating the vector length between the *x*–*y* positions of the spheroid center point and the respective microwell center point. Each center point was obtained by the “Centroid” function of ImageJ. Phase‐contrast micrographs of spheroids were analyzed, and spheroid diameters were measured on respective times using Olympus’ CellSense Standard software. To normalize fluorescent micrographs, Image backgrounds were subtracted, and mean fluorescence intensities (sum of the fluorescent values of all the pixels in the selection divided by the number of pixels) of spheroids were measured. In the case of penetration studies of doxorubicin, rhodamine123 and 4kDa FITC‐dextran, fluorescent values of the spheroid's core (150–100 µm from the edge) were measured. The Z‐stack images of single immunofluorescent stained BBB spheroids were obtained using an Olympus IX83 live‐cell microscope at 40× magnification. Z‐stacks of optical sections were captured across the entire spheroid thickness using excitation and emission (DAPI 350/470 nm, TRITC: 530/645 nm) settings for simultaneous dual‐channel recordings; approximately 20 Z‐stacks per spheroid were taken. Z‐stacks were processed and analyzed using the Wiener deconvolution by Olympus’ CellSense Standard software.

##### Statistical Analysis

All experiments were carried out at *n* = 3–12; exact numbers are mentioned per experiment in figure captions. For statistical analysis, data sets were tested for significance using Prism software 8 (Version 8.2.1; GraphPad, USA). Statistical analysis between three or more conditions was performed using the Mixed‐effects model, one‐way ANOVA, Holm‐Sidak's multiple comparisons test, Dunnett's multiple comparisons test, or analysis of covariance (ANCOVA). *P* values <0.0332 were considered as statistically significant (*p < 0.0332, **p < 0.0021, ***p < 0002, ****p < 0.0001). The data are presented as the mean ± standard deviation (SD).

## Conflict of Interest

The authors declare no conflict of interest.

## Author Contributions

C.E., M.R., F.S., and A.G. contributed equally to this work. C.E., M.R., F.S., A.G., and W.N. conceived and planned the experiments. C.J. and M.H. planned and carried out the simulations. C.E., F.S., A.G., and B.S. performed the experiments. C.E. analyzed the data and M.R., F.S., A.G., W.N., J.G., S.K., J.W., and P.E. contributed to the interpretation of the results. C.E., M.R., and P.E. wrote the paper with input from all authors.

## Supporting information

Supporting Information

## Data Availability

Research data are not shared.
