# Supplementary material for: A Microfluidic Multisize Spheroid Array for Multiparametric Screening of Anticancer Drugs and Blood–Brain Barrier Transport Properties
Source: Adv Sci (Weinh). 2021 Mar 24;8(11):2004856. doi: 10.1002/advs.202004856 (PMC8188192; doi:10.1002/advs.202004856)
Supplement: Supplementary file 1 — Supporting Information [file ADVS-8-2004856-s001.pdf]

© 2021 Wiley-VCH GmbH

ADVANCED SCIENCE
Open Access

Supporting Information

for *Adv. Sci.,* DOI: 10.1002/advs.202004856

A microfluidic multi-size spheroid array for multi-parametric screening of anti-cancer drugs and blood-brain barrier transport properties

Christoph Eilenberger, Mario Rothbauer, Florian Selinger, Anna Gerhartl, Christian Jordan, Michael Harasek, Barbara Schädl, Johannes Grillari, Julian Weghuber, Winfried Neuhaus, Seta Küpcü and Peter Ertl**

Supporting Information

A microfluidic multi-size spheroid array for multi-parametric screening of anti-cancer drugs and blood-brain barrier transport properties

Christoph Eilenberger, Mario Rothbauer, Florian Selinger, Anna Gerhartl, Christian Jordan, Michael Harasek, Barbara Schädl, Johannes Grillari, Julian Weghuber, Winfried Neuhaus, Seta Küpcü and Peter Ertl**

*corresponding authors: peter.ertl@tuwien.ac.at, mario.rothbauer@meduniwien.ac.at

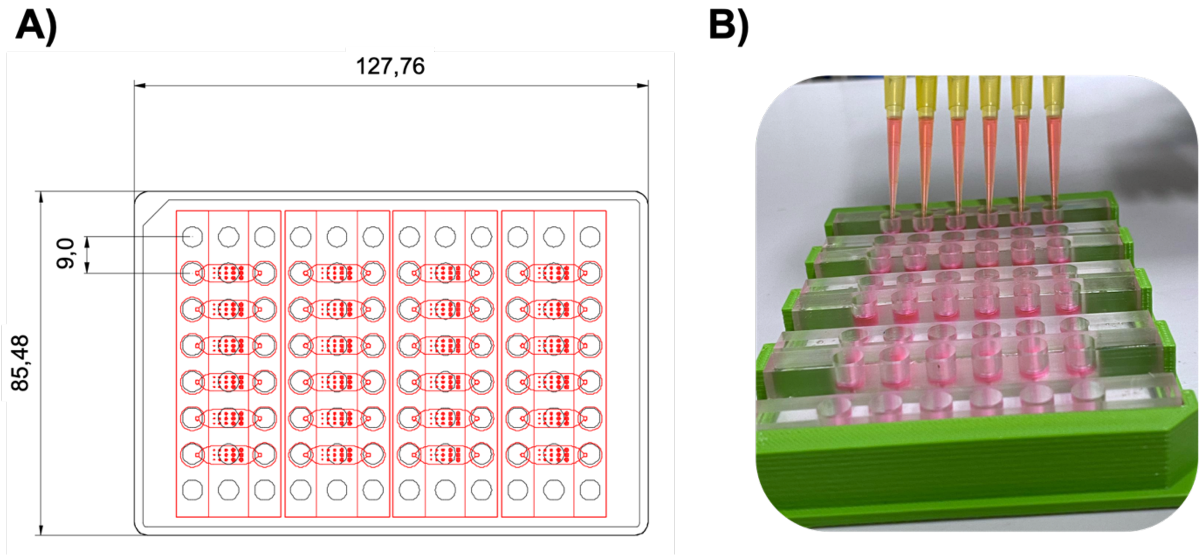

Figure SI-1: A) Design overlay of a standard 96-well microtiter plate and a panel of 4 microfluidic spheroid arrays positioned in the chip-frame showing the arrangement of media reservoirs and channel structures at a pitch of 9 mm and fitted widths of microchannels and reservoirs to microtiter well dimensions (in mm). **B)** Media reservoirs are conveniently addressable with standard multichannel pipettes and capable to generate 360 spheroids of 5 sizes in one microtiter plate design.

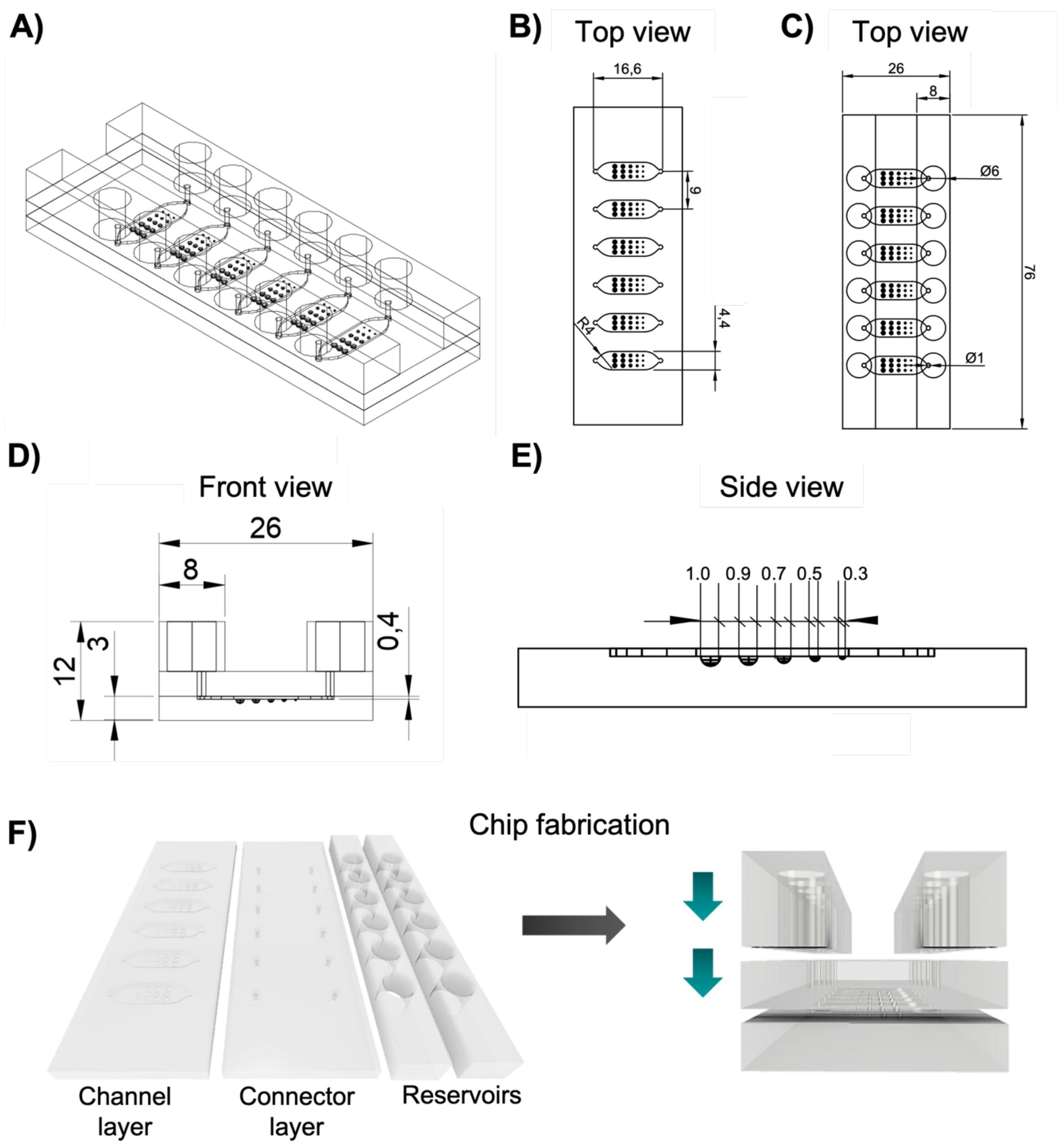

Figure SI-2: **A)** Three-dimensional graphical illustration of the microfluidic spheroid array including hemispherical microwells of different diameters for spheroid generation on the bottom, microfluidic connector holes and media reservoirs on the top. Engineering drafts of **B)** the top view of the entire device with media reservoirs, **C)** top view of the open channel, **D)** front view of the chip with respective heights of microchannels, micro connectors and reservoirs, and **E)** side view of the channel layer showing individual microwell diameters. All units are presented in mm. **F)** The platform comprises the microfluidic channel structure, a cover layers consisting of twelve connecting holes which are fluidically coupled to the reservoir layer ensuring continuous media perfusion.

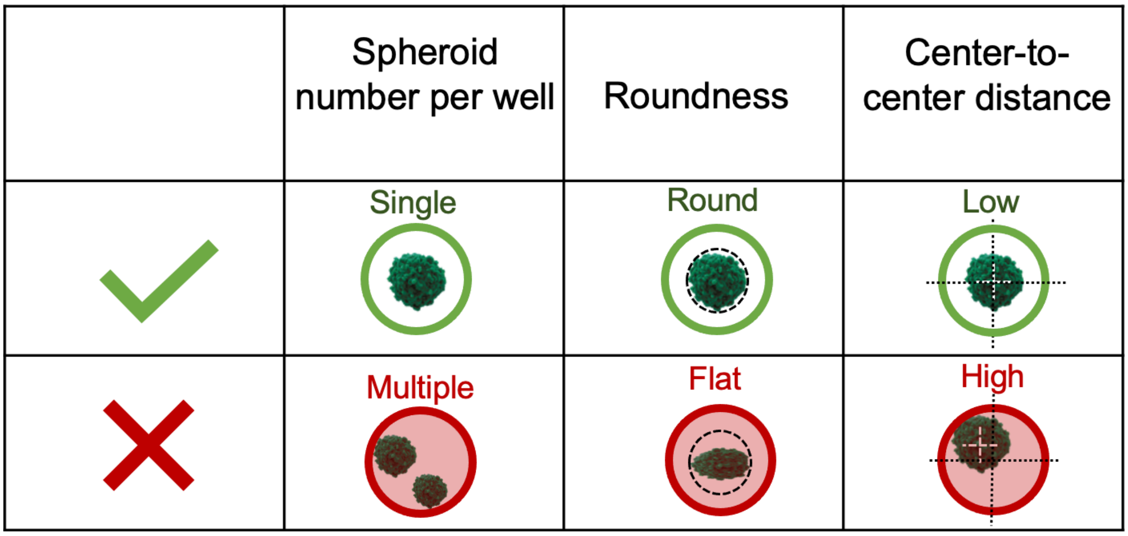

Figure SI-4: Schematic overview of the most optimal (green) and suboptimal (red) microwell quality parameters for reproducible spheroid generation and cultivation on-chip. Microwell dimensions were evaluated regarding number of spheroids per well, spheroid roundness, and spheroid center-to-microwell center-distances.

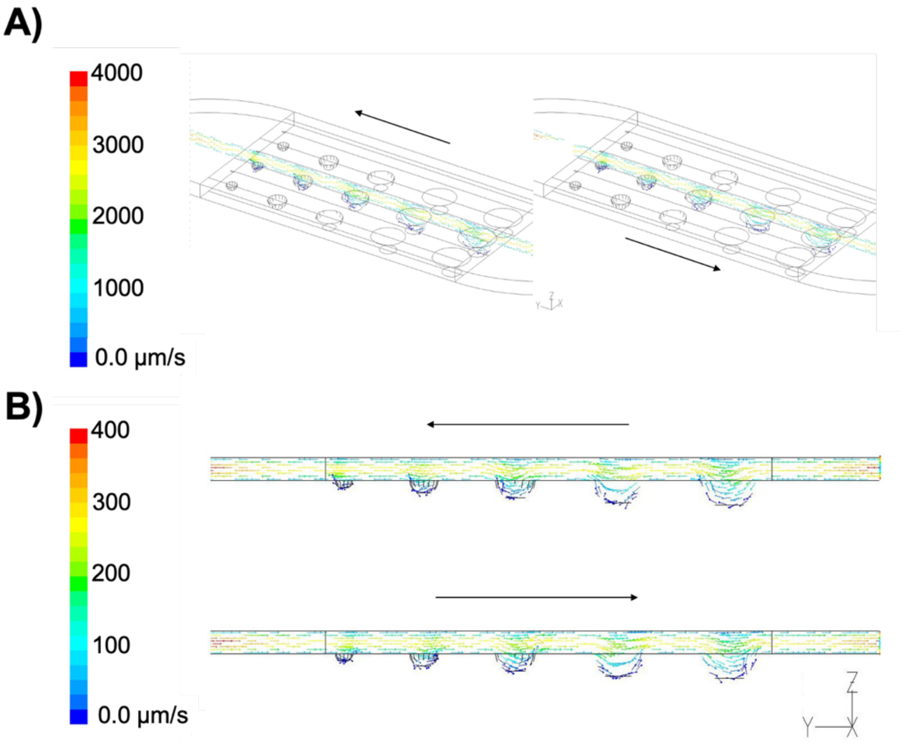

Figure SI-3: A) Top view and **B)** side view of flow velocity vector streams in spheroid array culture channels and microwells during tilting at an inclination angle of 1° and a speed of 1 rpm. Simulation was performed CFD (computational fluid dynamics) modelling.

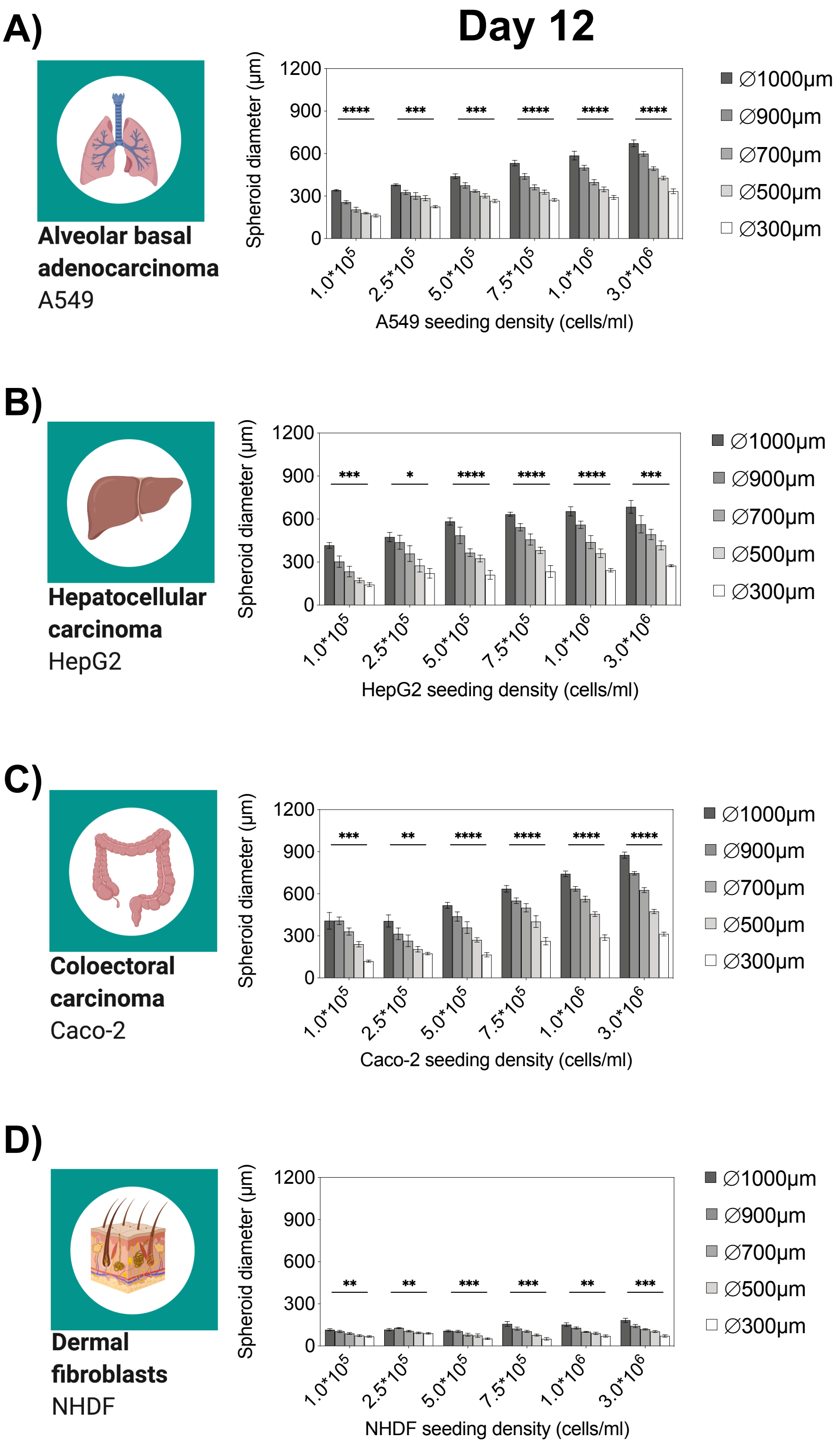

Figure SI-5: Analysis of spheroid diameters of **A)** A549, **B)** HepG2**, C)** Caco-2 and **D)** NHDF spheroids at different initial seeding densities over a cultivation period of twelve days under continuous perfusion, n=6-9 ± SD Statistical analysis was performed using Mixed-effects analysis (*p<0.0332, **p<0.0021, ***p<0.0002, ****p<0.0001).

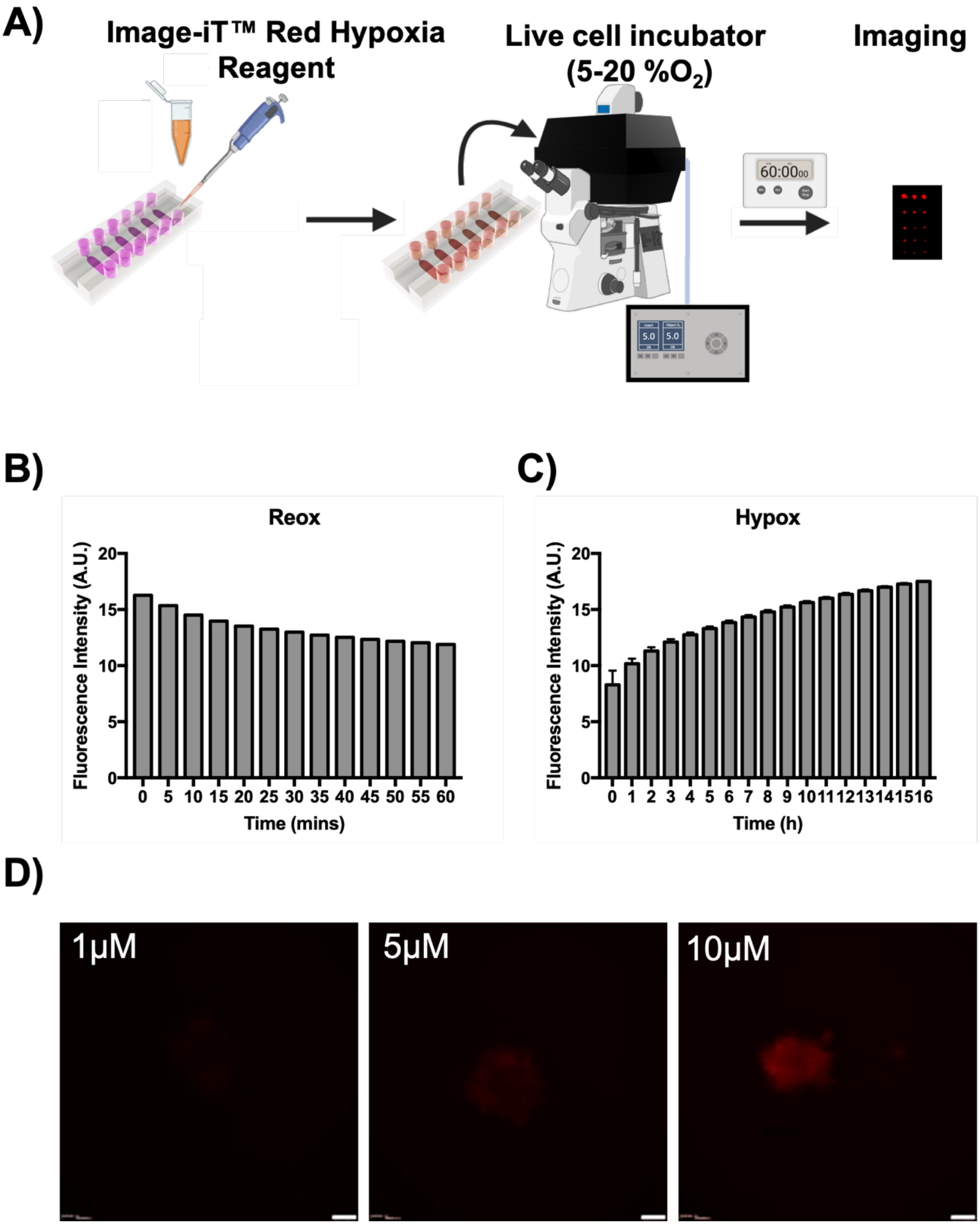
A)
Image-iT™ Red Hypoxia Reagent
Live cell incubator (5-20 %O2)
Imaging
60:00
5.0
5.0
B)
C)
Reox
Fluorescence Intensity (A.U.)
20
15
10
5
0
0 5 10 15 20 25 30 35 40 45 50 55 60
Time (mins)
Hypox
Fluorescence Intensity (A.U.)
20
15
10
5
0
0 1 2 3 4 5 6 7 8 9 10 11 12 13 14 15 16
Time (h)
D)
1μM
5μM
10μM

Figure SI-6: A) Experimental design of spheroid hypoxia imaging with the Image-iT™ Red Hypoxia Reagent (Invitrogen). The compound responds to increasing HIF-1alpha expression levels, appearing non-fluorescent when live cells are in an environment with normal oxygen concentrations and becomes fluorescent when oxygen levels are decreased. First, cell culture media was removed from reservoirs and and 200µl of Hypoxia reagent was added and incubated for 1 hour in live cell incubator. After 1 hour of incubation, hypoxia intensities were imaged using TRITC (Ex: 540/Em: 605) fluorescence filter. To first test the reagent sensitivity to low oxygen states, a 2D-monolayer culture of A549 cells stained with Image-iT Red Hypoxia Reagent in live cell incubator with varying oxygen levels. The graphs show the cellular response under **b)** low oxygen conditions at 5% O_2 and **c)** after restoring normal oxygen levels to 20% O_2. The monolayer cultures reacted to low oxygen conditions with a steep increase in signal emission within the first hour. After one hour of exposure, the hypoxia levels increase did not continue with this rate, n=3 ± SD. The declining rate indicates the approach of a plateau state after approximately 24 hours. Restoring oxygen levels (20% O_2) showed a decline in fluorescence intensity by a factor of about 1.4 AU within one hour. This validates that the read-out is sensitive to the decline of HIF-1a expression. **d)** For the determination of the optimal working concentration, A549 cells were seeded at a concentration of 10^4 cells/ml in an ultra-low attachment plate and cultivated at 37°C with 5% CO_2. After 7 days post-seeding A549 spheroids were treated with 1 µM, 5 µM and 10 µM of Image-iT. Red Hypoxia reagent for 1 hour. Scale bar, 200 µm. The hypoxia reagent applied to A549 spheroids showed the best results at a concentration of 10 µm. HIF-1a expression level appears to be low in spheroids under normal oxygen conditions leading to the requirement of higher reagent concentrations.

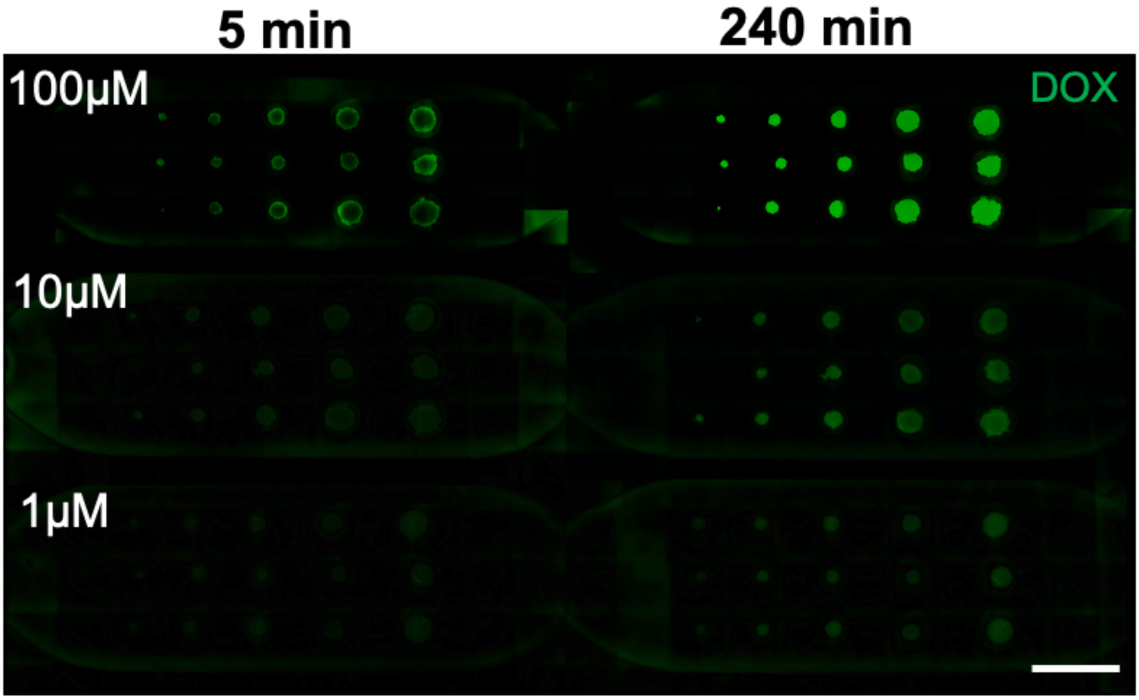

Figure SI-7: Fluorescent micrographs of treated A549 spheroids with the auto-fluorescent anti-cancer drug doxorubicin (DOX) at concentrations of 100 μM, 10 μM and 1 μM for an incubation spheroid of 240 minutes. Scale bar, 2 cm.

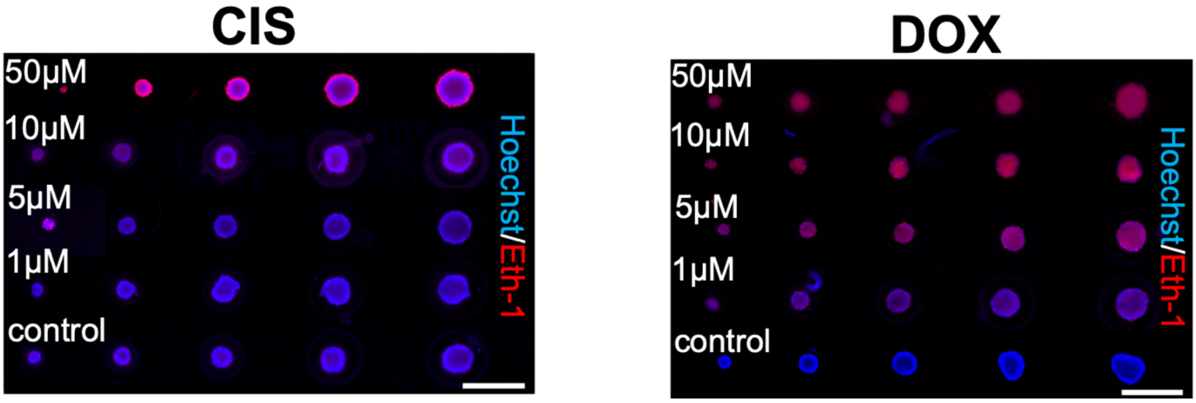

Figure SI-8: Fluorescent micrographs of treated different-sized A549 spheroids in the spheroid array chip with various doses of cisplatin (CIS) and doxorubicin (DOX)) for 24 hours to screen drug toxicity by staining cell nuclei (Hoechst; blue) and dead cells (Ethidium homodimer-1; red. Scale bar, 1 mm.

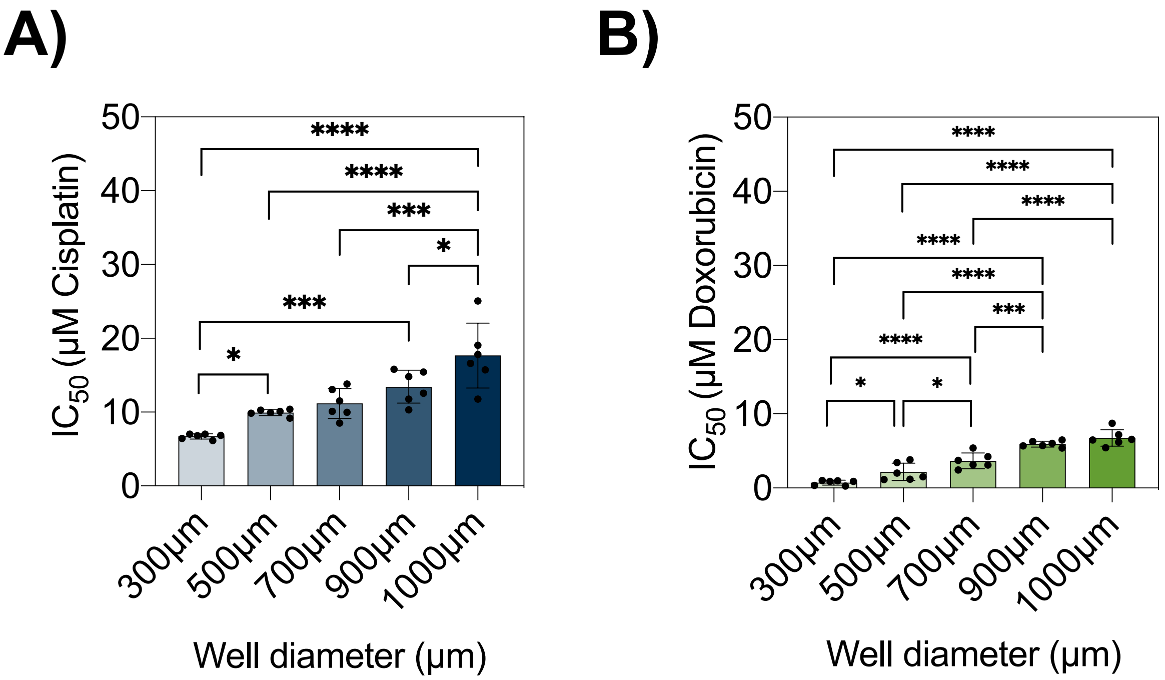

Figure SI-9: Comparative analysis of spheroid size-related effects on IC_{50} values of **A)** cisplatin and **B)** doxorubicin treated A549 spheroids, n=6 ± SD. Statistical analysis was performed using the Holm-Sidak's multiple comparisons test (*p<0.0332, **p<0.0021, ***p<0002, ****p<0.0001).

Table SI-1: Statistical analysis of combinatorial drug screening including cisplatin (CIS) and doxorubicin (DOX) by using the Mixed-effect model, n=3-6 (*p<0.0332, **p<0.0021, ***p<0002, ****p<0.0001, ns, not significant).

CIS:DOX concentrations [µM]	p - value	Summary
500:0	<0.0001	****
500:0.1	<0.0001	****
100:1	<0.0001	****
50:5	<0.0001	****
25:10	0.0004	***
10:25	0.0064	**
5:50	0.1287	ns
1:100	0.1902	ns
0.1:500	0.0556	ns
0:500	0.4062	ns

Table SI-2: Seeding densities of respective BBB cell ratios including human primary astrocytes (hA), human primary pericytes (hP) and hCMEC/D3 (BEC).

Total [cells/ml]	hA	hP	BEC
5.000.000	1	1	3
	1.000.000	1.000.000	3.000.000
5.000.000	1	1	2
	1.250.000	1.250.000	2.500.000
5.000.000	1	1	1
	1.666.667	1.666.667	1.666.667
5.000.000	5.5	1.5	3
	2.750.000	750.000	1.500.00
5.000.000	1	0	0
	5.000.000	0	0
5.000.000	1	4	0
	1.000.000	4.000.000	0

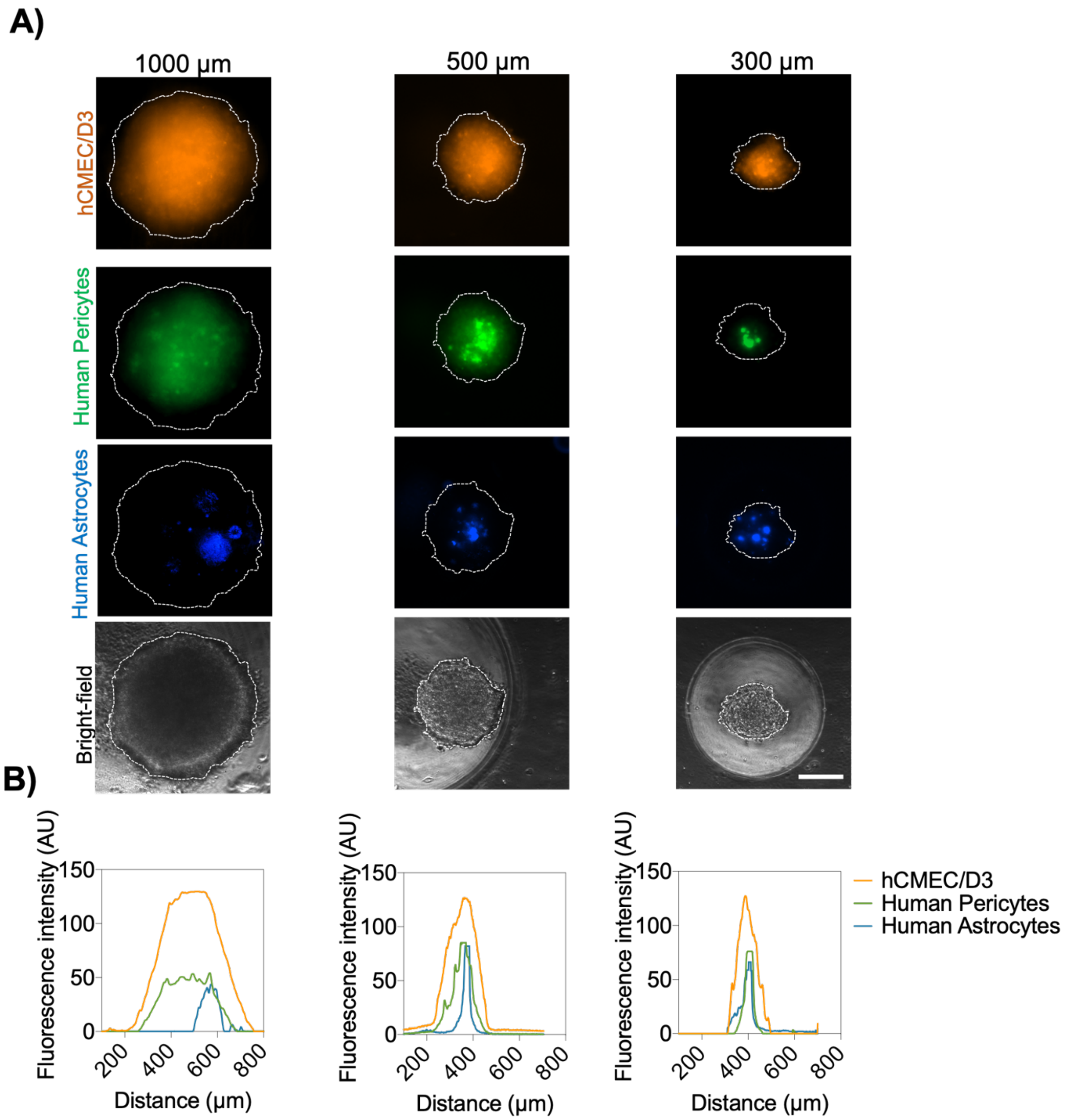

Figure SI-10: A) Fluorescent images of the internal organization of human brain endothelial cells (hCMEC/D3; orange), human pericytes (green), and human astrocytes (blue), when co-cultured to form spheroids after 6-days post-seeding at a cell ratio of 1:1:3 (hA:hP:BEC). Scale bar, 200 µm. **B)** Fluorescent intensity profiles of each labeled cell type in BBB triple-culture spheroids cultivated in 1000 µm, 500 µm, and 300 µm microwells on-chip under continuous perfusion.

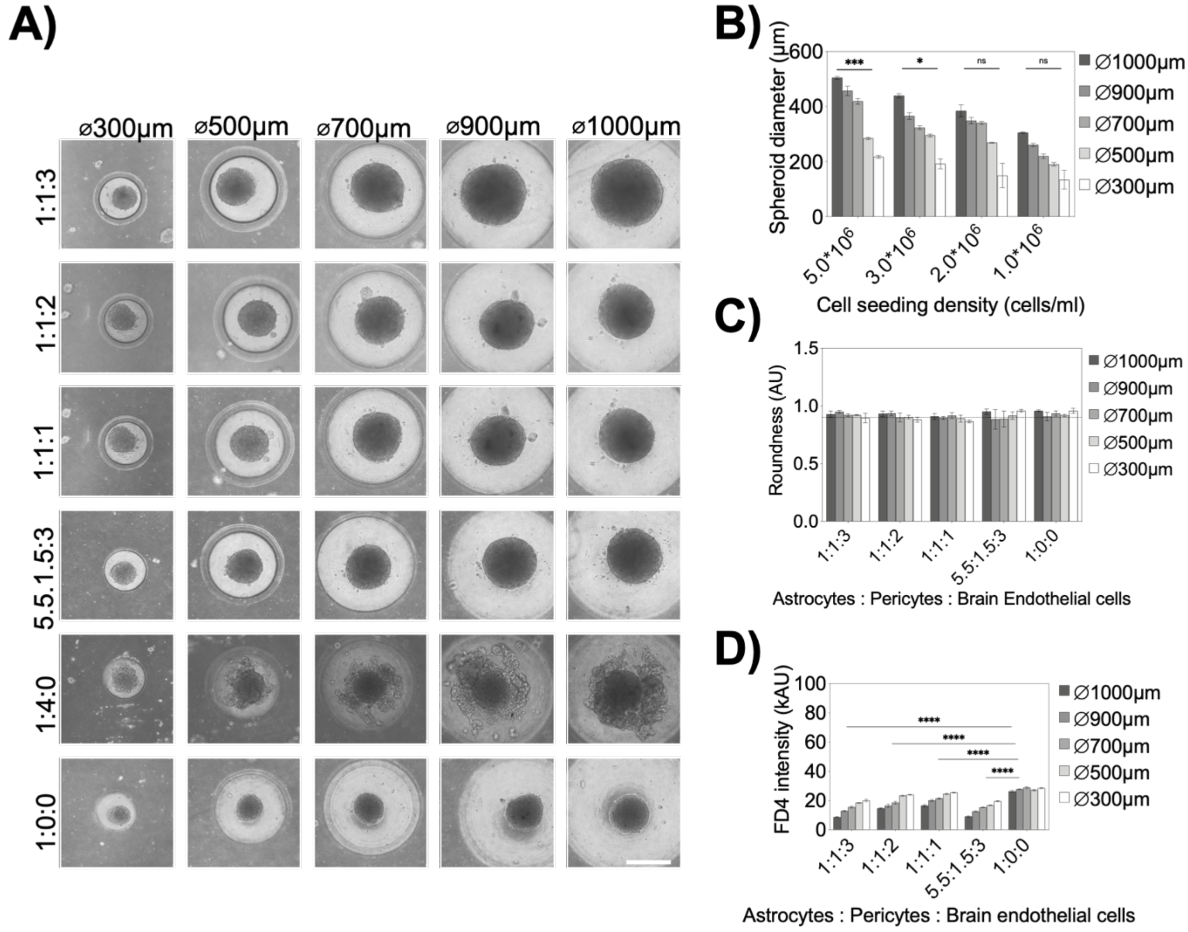

Figure SI-11: A) Bright-field micrographs of BBB spheroids after 6 days post-seeding at an initial cell density of $5*10^6$ cells/ml at different cell ratios of human primary astrocytes: human primary pericytes: human brain endothelial cells. Scale bar, 500 µm. **B)** Optimization of initial seeding density for on-chip spheroid co-culture generation regarding spheroid diameters at a ratio of 1:1:3 (hA:hP:BEC), n=3-6 ± SD. Statistical analysis was performed by using the Mixed-effects model. **C)** Roundness of BBB spheroids at different spheroid diameters and seeding ratios, n=3-6 ± SD. **D)** Mean fluorescence intensities of co-culture spheroids of different sizes and cell ratios after one hour of cultivation with 10 µM 4kDa FITC-Dextran (FD4), n = 7-9 ± SD. Statistical analysis was performed by using Dunnett's multiple comparisons test. (*p<0.0332, **p<0.0021, ***p<0.0002, ****p<0.0001, ns=not significant).
